# A whole-body atlas of BMP signaling activity in an adult sea anemone

**DOI:** 10.1186/s12915-025-02150-w

**Published:** 2025-02-21

**Authors:** Paul Knabl, David Mörsdorf, Grigory Genikhovich

**Affiliations:** 1 Department of Neurosciences and Developmental Biology, University of Vienna, Vienna, Austria; 2 Vienna Doctoral School of Ecology and Evolution (VDSEE), University of Vienna, Vienna, Austria

**Keywords:** BMP signaling, Cnidaria, *Nematostella*, *Tripedalia*, *Aurelia*

## Abstract

**Background:**

BMP signaling is responsible for the second body axis patterning in Bilateria and in the bilaterally symmetric members of the bilaterian sister clade Cnidaria—corals and sea anemones. However, medusozoan cnidarians (jellyfish, hydroids) are radially symmetric, and yet their genomes contain BMP signaling components. This evolutionary conservation suggests that BMP signaling must have other functions not related to axial patterning, which keeps BMP signaling components under selective pressure.

**Results:**

To find out what these functions might be, we generated a detailed whole-body atlas of BMP activity in the sea anemone *Nematostella*. In the adult polyp, we discover an unexpected diversity of domains with BMP signaling activity, which is especially prominent in the head, as well as across the neuro-muscular and reproductive parts of the gastrodermis. In accordance, analysis of two medusozoan species, the true jellyfish *Aurelia* and the box jellyfish *Tripedalia*, revealed similarly broad and diverse BMP activity.

**Conclusions:**

Our study reveals multiple, distinct domains of BMP signaling in Anthozoa and Medusozoa, supporting the versatile nature of the BMP pathway across Cnidaria. Most prominently, BMP signaling appears to be involved in tentacle formation, neuronal development, and gameto- or gonadogenesis.

**Supplementary Information:**

The online version contains supplementary material available at 10.1186/s12915-025-02150-w.

## Background

**Fig. 1:**
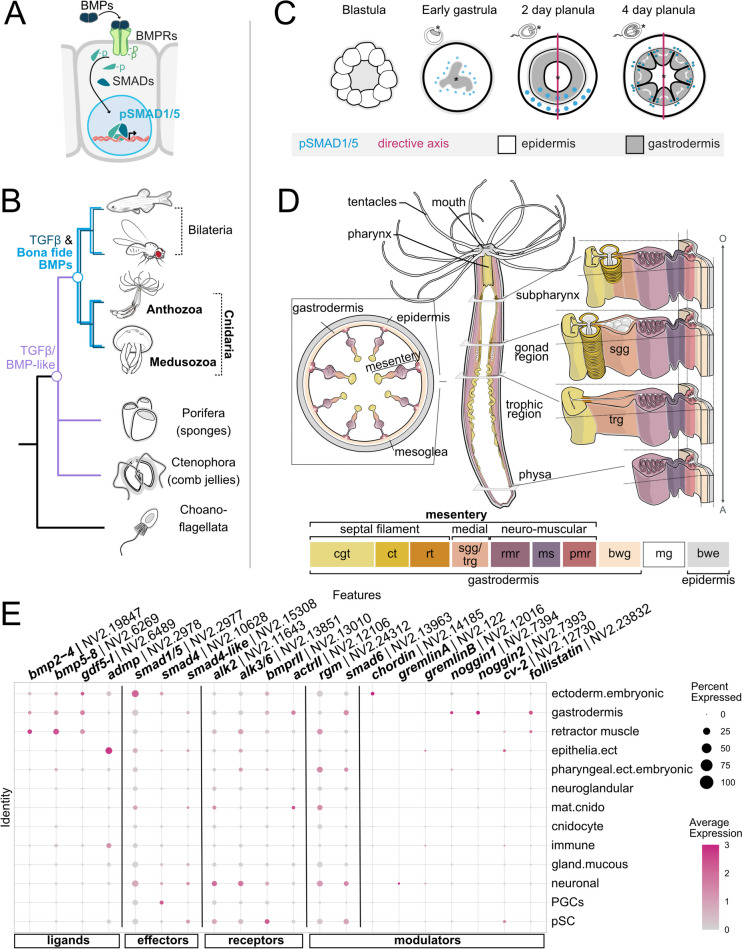
BMP pathway genes are evolutionary conserved between anthozoan Cnidaria and Medusozoa. **A** Activation of BMP signaling results in the phosphorylation of effector SMAD1/5 and its nuclear translocation to regulate BMP target gene transcription. **B** While TGFβ molecules are metazoan-specific, bona fide BMP genes are conserved between Bilateria and Cnidaria. **C** Dynamics of BMP signaling during early development of the sea anemone *Nematostella vectensis*. **D** Schematic of sea anemone *Nematostella* adult polyp highlighting mesentery anatomy at different body levels. bwe—body wall epidermis, bwg—body wall gastrodermis, mg—mesoglea, pmr—parietal muscle region, ms—mesentery stalk, rmr—retractor muscle region, sgg—somatic gonad gastrodermis, trg—trophic gastrodermis, rt—reticular tract, ct—ciliated tract, cgt—cnidoglandular tract. **E** Seurat dot plot visualizing the expression of BMP pathway genes in single-cell transcriptomic data of *Nematostella* adult polyp for coarse, tissue-level clustering [18]. Percent Expressed—dot size indicates the percentage of cells in a cluster expressing the gene of interest, Average Expression—color indicates averaged expression value

BMP signaling is initiated by the binding of dimeric BMP ligands to a tetrameric BMP receptor complex of two type II and two type I receptors. The receptor complex recruits and phosphorylates the transcriptional effector SMAD1/5 that binds the common-mediator SMAD4 to form a trimeric complex of two SMAD1/5 molecules and one SMAD4. The SMAD complex translocates to the nucleus, where it modulates BMP-responsive gene expression (Fig. 1A). Since the initial discovery of BMPs in the context of bone and cartilage morphogenesis [1, 2], BMP signaling has been studied in various vertebrate and invertebrate species, revealing far more versatile roles of the pathway. BMP signaling has been demonstrated to be involved in processes related to tissue homeostasis [3], apoptosis [4–6], cell fate commitment [7, 8], as well as gametogenesis, gonadogenesis, and reproduction [9–11]. Moreover, BMP signaling is vital for instructing pattern formation of various tissues and organs, such as the limb bud [12], the insect imaginal discs [13], the gastrointestinal tract [14, 15], the nervous system [8], and arguably most famously, the dorsoventral body axis across Bilateria [16, 17]. While the roles of BMP signaling in Bilateria are multifaceted, its functions in non-bilaterian Metazoa are much less explored.

BMP pathway members are not exclusive to Bilateria but highly conserved in the bilaterian sister clade Cnidaria, a phylum comprising Anthozoa (sea anemones and corals) and Medusozoa (jellyfish and hydroids) (Fig. 1B), the latter encompassing Hydrozoa (hydroids), Staurozoa (stalked jellyfish), Scyphozoa (true jellyfish), and Cubozoa (box jellyfish). All cnidarian classes possess the full complement of the intracellular BMP machinery, consisting of *bona fide* BMP ligands, receptors, and effectors [19–25]. Most of our knowledge about cnidarian BMP signaling to-date stems from Anthozoa, where it has been mainly studied in the sea anemone *Nematostella vectensis* and only in the context of axial patterning*.* Similar to Bilateria, *Nematostella*, and other Anthozoa form a bilaterally symmetric body plan with a secondary, “directive” body axis regulated by a BMP signaling gradient [26–28]. In the embryo, graded BMP signaling was shown to be responsible for the formation and patterning of the directive axis, requiring the interaction of multiple BMP signaling components. Knockdown of BMP signaling pathway members including *bmp2/4*,* bmp5-8*,* gdf5-like* (= *gdf5-l*),* chordin*,* gremlin*, and *rgm* resulted in defects or complete loss of the directive axis and, consequently, impaired patterning of the gastrodermal body layer^1^ [26–29]. The dynamics of BMP signaling have been studied during early *Nematostella* development, using an antibody against the phosphorylated form of the transcriptional effector of BMP signaling, pSMAD1/5 [26–28]. In the *Nematostella* late gastrula, pSMAD1/5 forms a bilaterally symmetric activity gradient, which disappears in the late planula larva (4 dpf = days post-fertilization) once the directive axis is established and patterned [27] (Fig. 1C). In the 4 dpf *Nematostella* planula, BMP signaling activity becomes radially symmetric and localizes to the oral epidermis, as well as to the newly formed gastrodermal folds termed the mesenteries [27] (Fig. 1C). To this point, it remains undetermined if and to which extent BMP signaling is required in other developmental processes beyond larval development of *Nematostella*.

Unlike bilaterally symmetric Anthozoa, Medusozoa lack the directive body axis and feature a radially symmetric body plan.^2^ Expression and transcriptomic data suggest that BMP signaling may be active in different medusozoan life stages, yet it is unknown which processes, if not the formation of a secondary axis, are regulated by BMP signaling in these animals. Previous analyses in several medusozoan species revealed generally broad expression profiles of BMP pathway genes across different body regions: The mRNA encoding the BMP ligand BMP5-8 has been found in the tentacle zone of the polyp of the scyphozoan *Aurelia* and the hydrozoans *Hydra*,* Podocoryne*, and *Clytia*, as well as the radial canals and sensory structures (rhopalia) in the *Aurelia* ephyra and in the medusae of *Clytia* and *Podocoryne* [23, 30–32]. In the hydroid *Hydractinia*, the expression of a putative *bmp receptor* was found in developing oocytes and in the male gonophore gastrodermis [33]. To our knowledge, in Medusozoa, the activity of BMP signaling has not yet been directly addressed by visualizing phosphorylated SMAD1/5 in any published work.

Given that BMP signaling remains active in the sea anemone after directive axis formation and that BMP components are present in medusozoan species lacking the secondary axis, we aimed to determine what roles other than directive axis patterning may be regulated by BMP signaling in Cnidaria. In this work, we analyzed the activity of BMP signaling under homeostatic conditions in several cnidarian species, with a special focus on the adult polyp of *Nematostella*. We analyzed the publicly available scRNA-seq data [18, 34, 35] and found a broad expression of BMP signaling components across many cell types. Then, using an antibody against the activated form of the BMP effector SMAD1/5, we generated a morphological atlas of pSMAD1/5 activity in the whole adult polyp of *Nematostella*, highlighting the surprisingly diverse activity domains across different body regions, especially in the inter-tentacle epidermis and specific portions of the mesentery gastrodermis (Fig. 1D). In the latter, we detected a population of pSMAD1/5-positive basiepithelial cells that, however, was distinct from previously described vasa2-positive basiepithelial stem-like cells. Together with a first characterization of BMP signaling in the jellyfish *Aurelia* and *Tripedalia*, we find evidence for the activity of BMP signaling in multiple cell types across different body regions, suggesting versatile roles of BMP signaling in Cnidaria.

## Results

### Single-cell RNA-seq data analysis suggests broad expression profiles of BMP pathway components across multiple cell populations in the adult *Nematostella* polyp

Thus far, expression studies of BMP pathway components and BMP signaling activity in *Nematostella* have been largely limited to early stages of development and the context of directive axis patterning [26–29, 36–39]. To get some information about where we can expect BMP signaling in the adult polyp, we interrogated the publicly available single-cell transcriptomic datasets for the expression of the BMP pathway genes [18, 34, 35]. Here, we focused on datasets of female and male polyps (“adult tissues”) with a coarse clustering approach recovering 13 distinct clusters based on the study by Cole et al. [18] (Fig. 1E). The *Nematostella* genome contains gene orthologues for four BMP ligands (*bmp2/4*, *bmp5-8*, *gdf5-l* and *admp* [19, 36, 38]), four BMP receptors (two type I receptors, *alk2* and *alk3/6* and two type II receptors, *bmprII* and *actRII*; Additional file 1:Fig. S1), and two BMP effectors (*smad1/5* and *smad4*, as well as a “truncated” *smad4-like* [38]). In addition, several orthologues of potential BMP signaling antagonists are present, including transmembrane (*rgm* [28]), intracellular (*smad6* [38]), and extracellular or secreted molecules (*chordin*, *gremlinA*, *gremlinB*, *noggin1, noggin2*, *crossveinless-2*, and *follistatin* [19, 37–39]; Additional file 1:Fig. S1).

BMP receptors and BMP effectors displayed low expression levels but with surprisingly broad expression profiles and transcripts detectable in multiple cell clusters. Intracellular components were present in mature cnidocytes (“mat.cnido”), “retractor muscle,” “gastrodermis,” epithelial ectoderm (“epithelia.ect”) and embryonic ectoderm (“ectoderm.embryonic”), and upregulated in putative stem cell (“pSC”), and neurosensory and neurosecretory (“neuronal”) cell types. In contrast, the expression of BMP ligands was spatially segregated, with *bmp2/4*, *bmp5-8*, and *gdf5-l* exhibiting similar expression profiles in the “gastrodermis,” “retractor muscle,” and “ectoderm.embryonic,” whereas *admp* expression was restricted to the “epidermis.” For the expression of BMP receptor types, no obvious cell type-specific segregation was apparent. Together, the expression of BMP pathway genes in the single-cell transcriptomic data suggests that BMP signaling is potentially active in various cell populations, involving few “BMP secreting” cell types that can signal to a wider range of “BMP receiving” cell types. BMP pathway genes display low expression levels and are limited to a low number of cells per cluster (transcripts expressed in < 16% of cells of each cluster), which might be due to diverging expression profiles within the different clusters. Indeed, expression across the 91 adult-restricted transcriptomic states shows that the expression of BMP pathway genes can vary between different sub-clusters in each coarse cluster (Additional file 1:Fig. S2).

### BMP signaling forms distinct activity domains in the inner and outer epithelial layers of the adult polyp

**Fig. 2:**
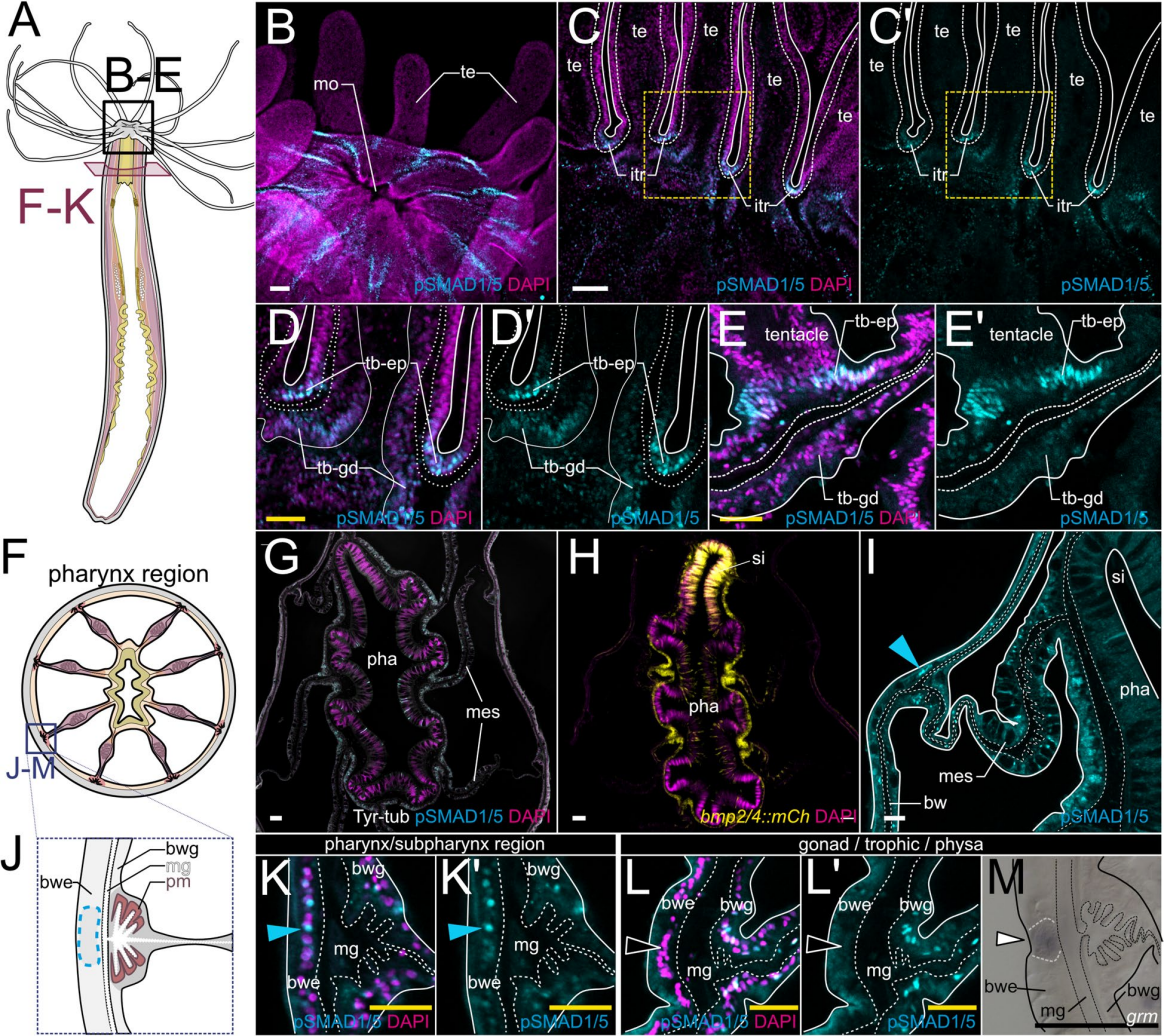
Epidermal BMP signaling is confined to the oral epithelium. Characterization of BMP signaling in the oral region by anti-pSMAD1/5 staining (cyan). DNA is stained with DAPI (magenta), outlines of tissue layers (solid white lines) and mesoglea (dotted white lines) are indicated. **A** Mouth region (black box), cross-section of the body column at the pharynx level (red box), **B** pSMAD1/5 staining in the region of the hypostome, **C** longitudinal section of the tentacle and tentacle base, **D,E** details of the tentacle base, **F** schematic of a cross-section at the pharynx level, **G** overview of a pharynx cross-section, **H** mCherry expression in the *bmp2/4::mCh* reporter line highlights the ciliated groove (siphonoglyph) on one side of the pharynx, **I** detail of a pharynx cross-section, pSMAD1/5-positive cells are located in the epidermis at the position of the mesentery attachment site (blue arrowhead), **J** schematic of the mesentery attachment site, **K–K’** pSMAD1/5-positive nuclei in the epidermis of the mesentery attachment site at the level of the pharynx and subpharynx, **L–L’** BMP signaling is absent in the respective regions at the level of the gonad, trophic, or physa region, **M** epidermal *gremlin* expression. mo—mouth, te—tentacle, itr—inter-tentacular region, tb—tentacle base, tb-ep tentacle base epidermis, tb-gd—tentacle base gastrodermis, pha—pharynx, si—siphonoglyph, mes—mesentery, mg—mesoglea, bwe—body wall epidermis, bwg—body wall gastrodermis. All stainings were performed at least two times independently with 3 or more animals imaged. Scale bars 50 µm (white), 25 µm (yellow), and 100 µm (purple)

To explore BMP signaling unrelated to axial patterning, we analyzed the activity of BMP signaling in the sexually mature polyp (Fig. 2A), utilizing the cross-reactive commercial antibody against the phosphorylated form of the BMP signaling effector SMAD1/5, which we have successfully used in *Nematostella* embryos previously [27]. In the adult, we detected distinct domains of pSMAD1/5 activity in both epithelial body layers (Fig. 1D), comprising the “outer” epidermis and the “inner” gastrodermis, which are separated by an extracellular matrix layer (mesoglea). Most of the epidermal epithelium was free of pSMAD1/5 activity, except for the oral domain (Fig. 2B, Additional file 1:Fig. S3). Here, BMP signaling was detectable in the inter-tentacular spaces (Fig. 2C–E’), as well as in longitudinal, epidermal stripes (Fig. 2I–L’). BMP signaling between the tentacles was present at each tentacle base with pSMAD1/5 activity mostly evident in the epidermis (Fig. 2C–E’), slightly reduced in the gastrodermis (Fig. 2D), and completely absent from other parts of the tentacles, except for the unspecific, non-nuclear staining of the capsules and threads of the tentacle-specific cnidocyte type—the spirocytes (Additional file 1: Fig. S4). In the heterozygous *bmp2/4::mCherry* transgenic animals, in which mCherry reporter is expressed under control of the 4.5-kb regulatory region upstream of the *bmp2/4* start codon [40], strong mCherry expression marks one side of the pharynx forming the ciliated groove, the siphonoglyph (Fig. 2F–H,Additional file 1:Fig. S5). Previous work also showed that *bmp2/4* mRNA is present in the siphonoglyph cells of the planula larva [36]; however, we did not detect above-background pSMAD1/5 staining in this area in the adult.

In the body wall epidermis, at the level of the pharynx, epidermal clusters of pSMAD1/5-positive cells assemble into eight longitudinal stripes (Fig. 2I–L’), correlating with the positions of each mesentery attachment site in the gastrodermis (Fig. 2J). Notably, discernible BMP signaling activity in the stripes was restricted to the pharyngeal and subpharyngeal region (Fig. 2K–K’), while absent from more aboral positions (Fig. 2L–L’). There, at the levels of the gonadal, trophic, and physa region, BMP antagonist *gremlin* was expressed in the epidermis *vis-à-vis* the mesentery attachment sites, and this area was free of pSMAD1/5 activity (Fig. 2M).

**Fig. 3:**
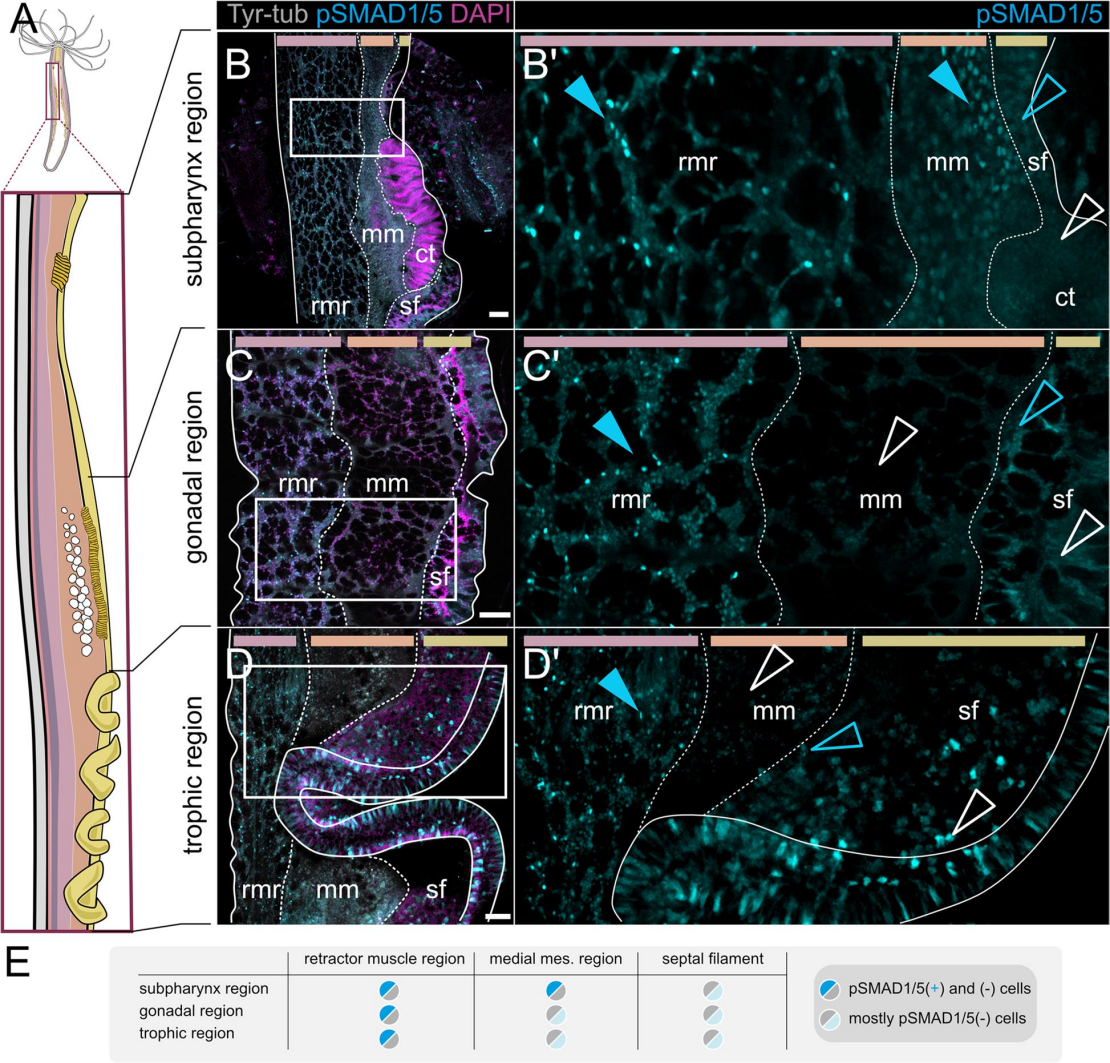
Different regions of the oral-aboral axis display different patterns of BMP signaling activity in the mesentery.** A** Sketch of the lateral overviews of mesenteries at different positions along the oral-aboral body axis shown on **B–D’**. White lines demarcate different mesentery domains, pSMAD1/5 (cyan), and DNA (magenta). **B** Subpharyngeal region displays pSMAD1/5 activity in the rmr (retractor muscle region) and mm (medial mesentery) (blue solid arrowheads), and few pSMAD1/5-positive nuclei in the cnidoglandular tract of the septal filament (sf; blue, hollow arrowhead), **C** gonadal region, and **D** trophic region, display pSMAD1/5 activity in the rmr, pSMAD1/5 activity is mostly absent in the mm (white, hollow arrowhead) and few pSMAD1/5-positive nuclei in the sf (blue, hollow arrowheads). **B'**, **C'** and **D'** show regions boxed in **B**, **C** and **D**, respectively. **E** Summary of BMP signaling domains across different mesentery regions. All stainings were performed at least two times independently with 3 or more animals imaged. Scale bars 50 µm. rmr—retractor muscle region, mm—medial mesentery, sf—septal filament, ct—ciliated tract

In contrast to the orally restricted BMP signaling in the epidermis, BMP signaling in the gastrodermis was evident along the entire primary, oral-aboral (OA) body axis (Fig. 3), and especially prominent in the mesenteries—the eight folds of the gastrodermal epithelium partitioning the gastric cavity (Figs. 1D, 3A), where pSMAD1/5 activity was highly pronounced. We did not observe any differences in pSMAD1/5 staining between different mesenteries, however, when comparing BMP signaling in the basal, medial, and apical portion of the mesentery, we observed differences in activity depending on the position along the OA body axis (Fig. 3A–D’). BMP signaling in the basal part of the mesentery (neuro-muscular mesentery, closest to the body wall) was the most consistent along the OA axis, with continuous and pronounced signaling activity from pharynx to physa (Fig. 3A–C’, blue solid arrowheads). The apical domain of the mesentery (septal filament, cnidoglandular tract) displayed little nuclear pSMAD1/5, but often showed unspecific cytoplasmic or vesicular signal. Unspecific signal was repeatedly observed in putative muco-glandular cells that are most abundant in the trophic region (Fig. 3C–C’, white arrowheads in the sf region; Additional file 1:Fig. S4). Unlike in the basal and apical domain, there were distinct differences in pSMAD1/5 staining in the medial mesentery portion, changing according to the body region along the primary body axis (Fig. 3A–D’, mm region). While BMP signaling was broadly active in the medial mesentery of the pharynx and upper subpharynx region (Fig. 3B–B’, solid blue arrowheads in the mm region), pSMAD1/5 was reduced or absent from the medial gastrodermis at lower positions (lower subpharynx region, gonad region, and trophic region) (Fig. 3C–D’, hollow white arrowheads in the mm region).

### Gastrodermal BMP signaling occurs in distinct domains along the basal–apical axis of the mesentery

**Fig. 4:**
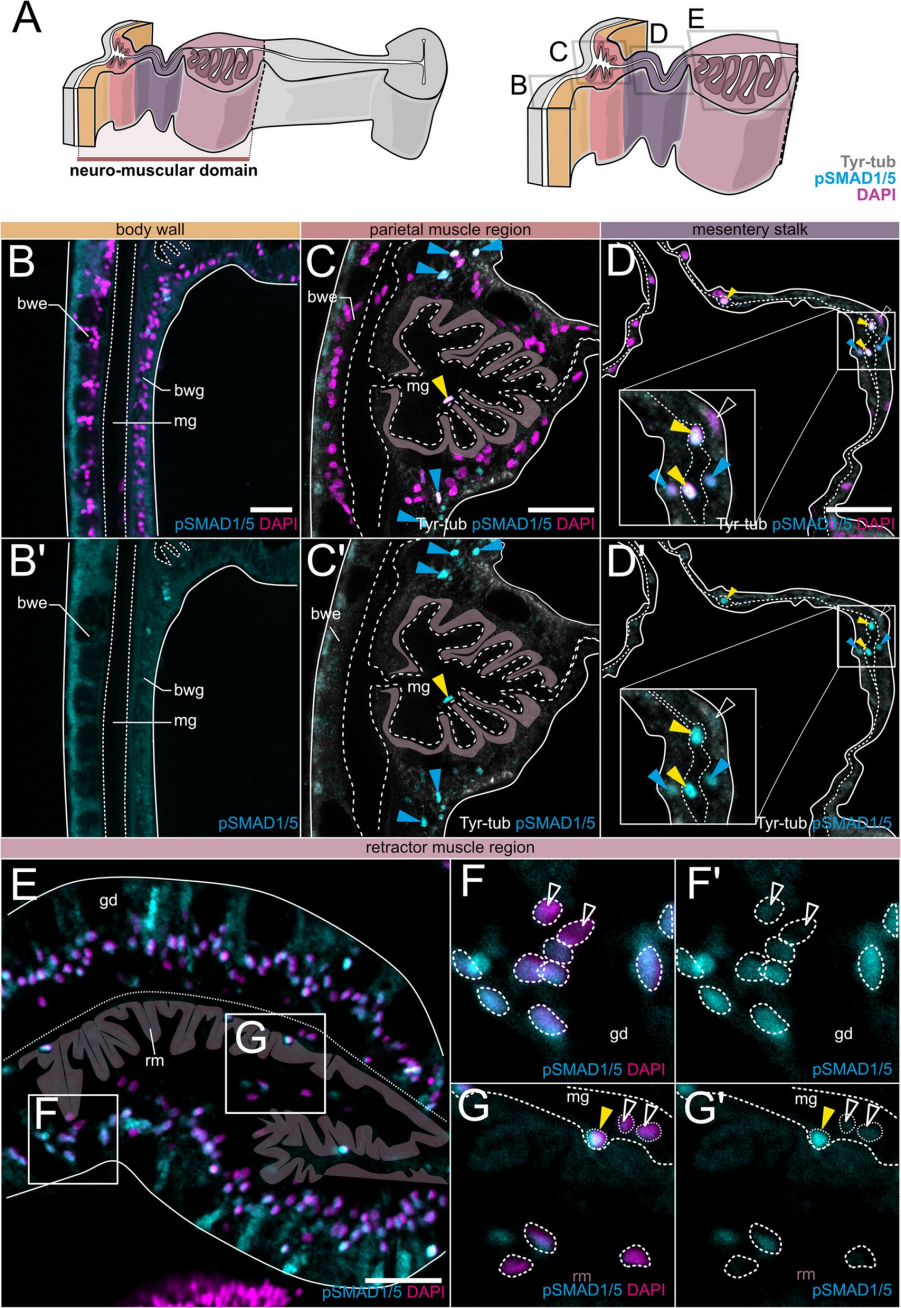
BMP signaling is active in epithelial and basiepithelial cells of the neuro-muscular mesentery. Immunostaining for pSMAD1/5 (cyan), Tyr-tubulin (gray), and DNA (magenta). **A** Schematic overview of a mesentery cross-section illustrating its morphological domains. Basal mesentery regions are highlighted in color. Images on **B–E** are transverse sections corresponding to regions boxed on **A**. **F–G’** are high magnification views of areas boxed on **E**. **B–B’** pSMAD1/5-negative body wall gastrodermis and epidermis. **C–C’** pSMAD1/5-positive epithelial cell clusters (blue arrowheads) and individual basiepithelial cells (yellow arrowheads). **D–D’** pSMAD1/5-positive and -negative cells in the epithelium and the mesoglea of the mesentery stalk (yellow arrowheads). **E** Cross-section of the retractor muscle region with pSMAD1/5-positive and pSMAD1/5-negative epithelial and basiepithelial cells, retractor muscle myonemes are highlighted in mauve. **F’–F”** Magnified view of the retractor muscle region epithelium with pSMAD1/5-positive and -negative gastrodermal cells. **G’–G”** A region of the retractor muscle with pSMAD1/5-positive and -negative epithelial and basiepithelial cells. All stainings were performed at least two times independently with 3 or more animals imaged. Scale bars 25 µm. mg—mesoglea, bwe—body wall epidermis, bwg—body wall gastrodermis, gd—gastrodermis, rm—retractor muscle

**Fig. 5:**
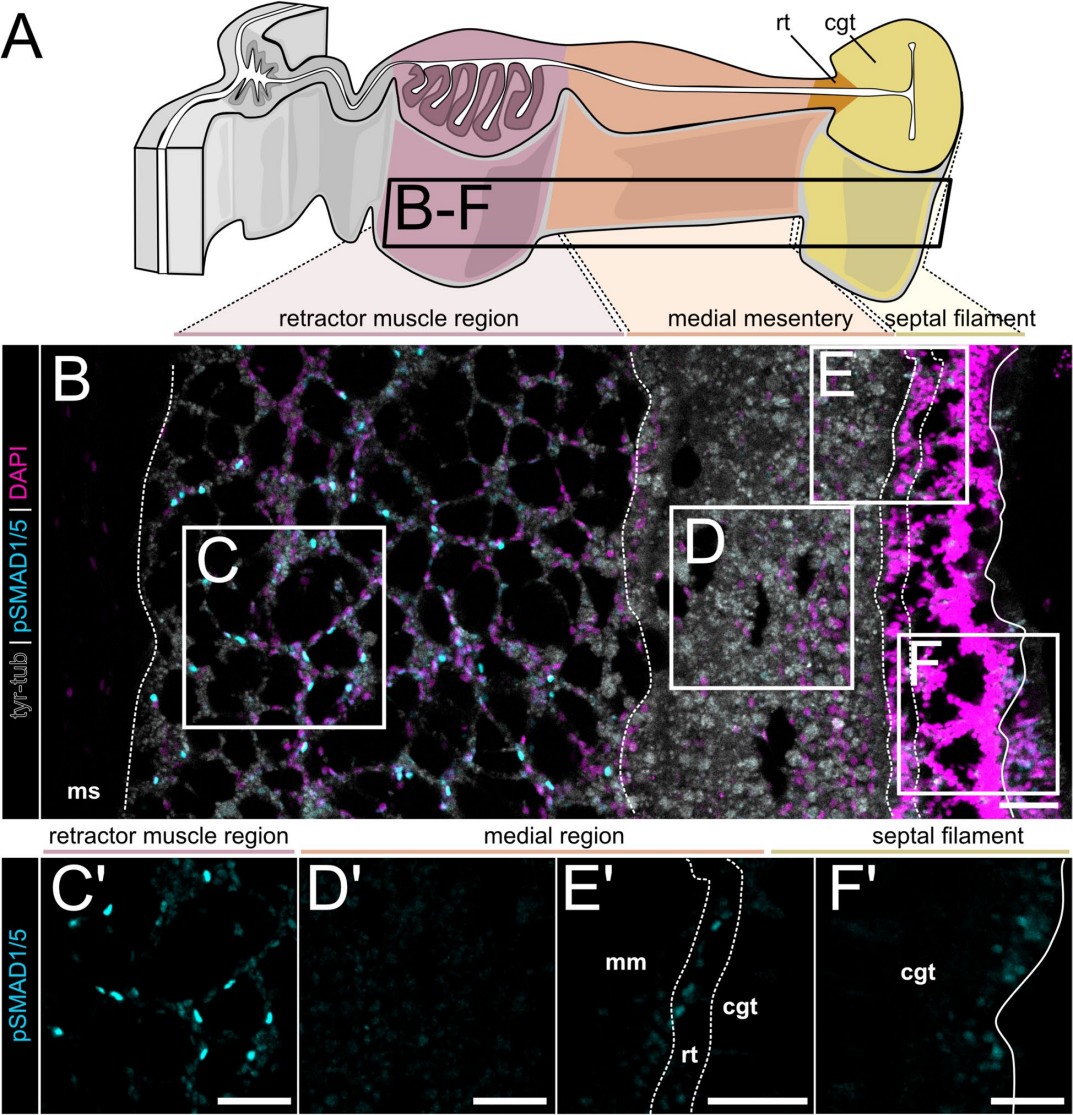
Differences in the BMP signaling domains of the medial and apical mesentery region. Immunostaining for pSMAD1/5 (cyan), Tyr-tubulin (gray), and DNA (magenta). **A** Schematic overview of a mesentery cross-section illustrating morphological domains, highlighted are the retractor muscle region, the medial and the apical (= septal filament) mesentery domains. **B** Lateral view of the mesentery at the level between subpharynx and gonad region. **C–C’** Broad pSMAD1/5 activity in the retractor muscle region. **D–D’** pSMAD1/5 is mostly absent from the medial mesentery. **E–E’** Individual pSMAD1/5-positive cells at the boundary of the medial mesentery and the septal filament in the reticular tract. **F–F’** Individual pSMAD1/5-positive cells in the cnidoglandular tract of the septal filament. All stainings were performed at least two times independently with 3 or more animals imaged. rmr—retractor muscle region, mm—medial mesentery, rt—reticular tract, cgt—cnidoglandular tract. Scale bars 50 µm

To get a more detailed picture of BMP signaling in different parts of the mesentery, we analyzed the pSMAD1/5 activity in the individual mesentery domains, starting at the body wall and the basal part of the mesentery (neuro-muscular domain, Fig. 4), moving along the medial mesentery domain and into the septal filament at the mesentery tip pointing towards the center of the gastric cavity (Fig. 5). In the mesenteries, we could differentiate between (1) pSMAD1/5-positive epithelial cell populations in different domains of the mesentery and (2) pSMAD1/5-positive interstitial or basiepithelial cells, located in the extracellular matrix (mesoglea).

The body wall gastrodermis between the mesenteries was generally free of BMP signaling (Fig. 4B–B’), although individual pSMAD1/5-positive cells were occasionally observed. At the base of each mesentery, BMP signaling was active in two epithelial cell clusters located on both sides of the parietal muscle (Fig. 4C–C’, blue arrowheads), while other cells of the parietal muscle region were pSMAD1/5-negative. The adjoining mesentery stalk contained pSMAD1/5-positive and -negative epithelial cells, distributed throughout the stalk (Fig. 4D–D’). The gastrodermal epithelium of the retractor muscle region displayed pronounced pSMAD1/5 activity, also with intermixed communities of pSMAD1/5-positive and pSMAD1/5-negative cells (Fig. 4E–G’).

Aside from epithelial cell populations, we also observed interstitial, basiepithelial cells that were present in the mesoglea of the mesentery and the body wall, across different locations of the body column, as previously described by others [41, 42]. Notably, many basiepithelial cells located in the parietal muscle region, along the mesentery stalk and in the retractor muscle region were pSMAD1/5-positive (Fig. 4C–D’, G–G’, yellow arrowheads), although we observed basiepithelial cells without BMP signaling activity in the same region as well. Strikingly, BMP signaling activity in these cells appeared to be restricted to the neuromuscular region of the mesentery but was absent from other portions of the mesentery or other locations of the body column. This indicates that there are different subpopulations of basiepithelial cells present in the mesoglea that can be either pSMAD1/5-positive or -negative, and/or that BMP signaling in these cells is only active in a specific context, which may possibly be linked to their differentiation choices.

In the medial and apical mesentery (Figs. 5A,Additional file 1:Fig. S6), activity domains of BMP signaling were less pronounced compared to the basal mesentery (Fig. 5B–F’). In the medial domain, BMP signaling was mostly absent (Fig. 5B, D’–E’), except at the levels of the pharynx and subpharynx (Fig. 3A–A’), as described above, and in a narrow stripe of cells along the periphery of the medial domain at its border to the reticular tract (rt) in the gonadal region or to the cnidoglandular tract (cgt) in the trophic region (Fig. 5E’). Other exceptions, observed in the somatic gonad, are outlined in a separate section below. The cnidoglandular tract was mostly devoid of BMP signaling; however, sporadically, pSMAD1/5-positive cells were also observed in different locations of the septal filament (Fig. 5F’, Additional file 1:Fig. S6).

Overall, we observed intermixed communities of pSMAD1/5-positive and -negative cells, with epithelial or basiepithelial localization, that form multiple BMP signaling domains in the mesentery. pSMAD1/5 activity domains of the basal, neuro-muscular mesentery are highly pronounced, compared to the more reduced or completely absent activity in the medial and apical mesentery. This heterogeneity in composition and location of pSMAD1/5 domains suggest that BMP signaling activity is not limited to a single BMP-responsive cell population but rather can be active in multiple cell types or cell states, which is supported by the broad expression of intracellular BMP components in the single-cell transcriptomic data.

### Medial and apical mesentery domains display reduced pSMAD1/5 activity but increased BMP expression

**Fig. 6:**
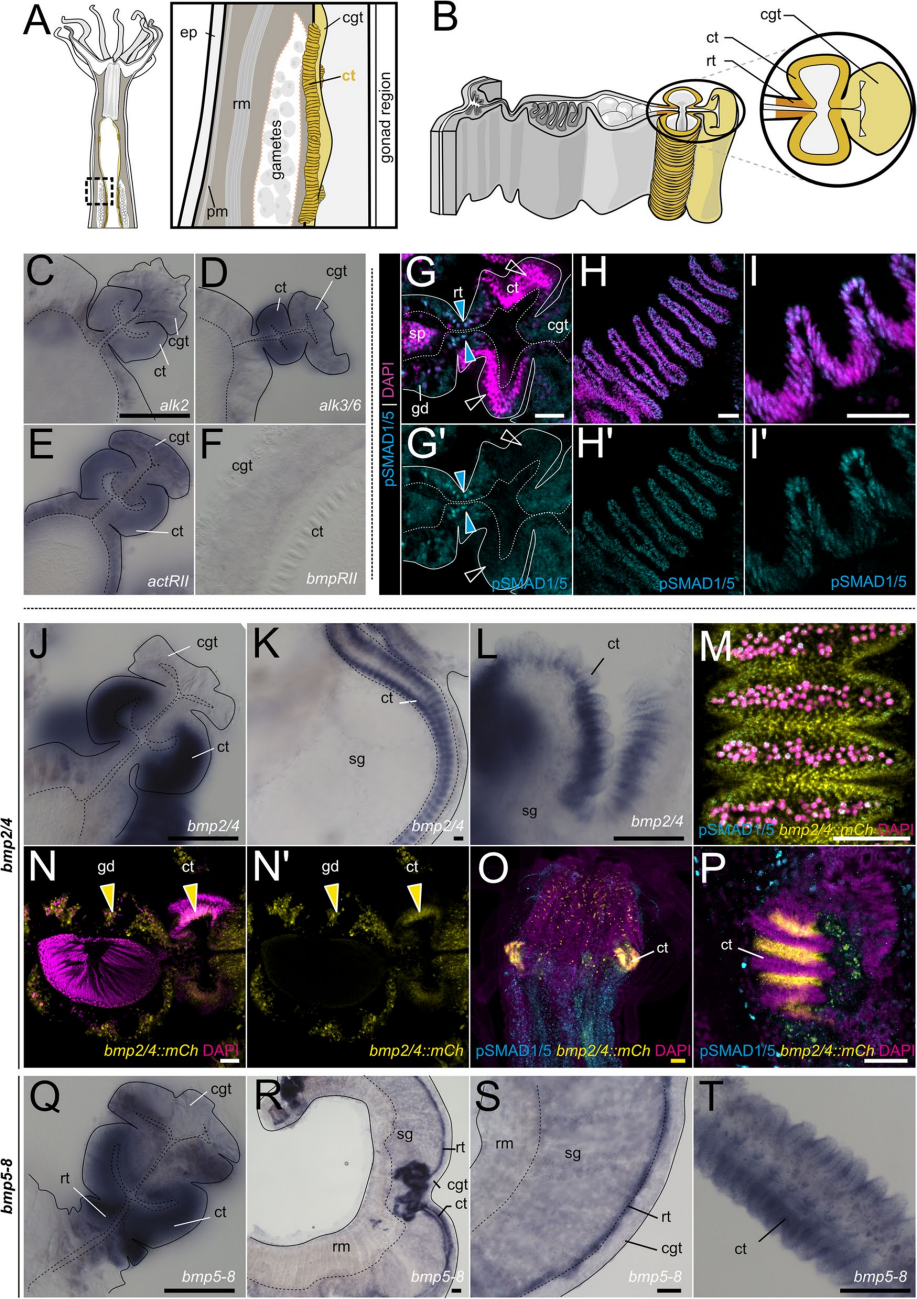
pSMAD1/5 activity, BMP, and BMP receptor expression in the septal filament.** A,B** Schematics of the mesentery morphology at the level of the gonad region in a lateral view (**A**) and in a transverse section (**B**). Sub-regions of the septal filament highlighted in shades of yellow. **C–F** BMP receptor expression of the type I receptors *alk2* (**C**) and *alk3/6* (**D**), and of the type II receptors *actRII* (**E**) and *bmprII* (**F**) in the septal filament. **G–I’** Immunostaining for pSMAD1/5 (cyan) with DNA counterstained in magenta in the septal filament. **G–G’** Cross-section of the mesentery of a male in the gonad region, ciliated tracts are indicated (ct), blue arrowheads highlight pSMAD1/5 in the reticular tract (rt), **H–H’** top view of the ciliated tract showing pSMAD1/5 activity in its folds. **I–I’** Longitudinal optical section of the ciliated tract showing pSMAD1/5 activity in the folds. **J–L** *bmp2/4* expression in the septal filament in cross-section and lateral views. **M–O** mCherry expression in the *bmp2/4::mCherry* reporter line. **M–N’** mCherry is expressed in the mesentery gastrodermis and in the ciliated tract of the adult polyp. **O–P** *bmp2/4::mCherry* activity in the ciliated tract in the juvenile polyp. **Q–T** *bmp5-8* expression in the mesentery gastrodermis, reticular tract and ciliated tract; cross-section (**Q**), and lateral views (**R-T**). All stainings were performed at least two times independently with 3 or more animals imaged. Scale bars 25 µm (white), 50 µm (yellow), and 100 µm (black). ep—epidermis, pm—parietal muscle, rm—retractor muscle, gd—gastrodermis, sp—spermary, rt—reticular tract, ct—ciliated tract, cgt—cnidoglandular tract, sg—somatic gonad

Our characterization of BMP signaling domains in the mesentery revealed the intriguing reduction of signaling activity in the medial and apical mesentery including the gonad region (Fig. 6A,B) that we aimed to address in more detail (Fig. 6,Additional file 1:Fig. S7). First, we analyzed the expression of BMP receptors using colorimetric in situ hybridization, and detected *alk2*, *alk3/6* and *actrII* but no *bmprII* mRNA in parts of the septal filament, with an upregulated expression in the reticular and ciliated tracts in the gonad region of the polyp (Fig. 6C–F). We also detected BMP signaling activity there, although pSMAD1/5 staining appeared weaker than in the areas described in the previous chapters. In the septal filament, BMP signaling was active in the epithelial cells of the reticular tract (Fig. 6G–G’, blue solid arrowheads), while it was absent from basiepithelial cells. Similarly, low levels of BMP signaling were sometimes detectable in the ciliated tract of the gonad region (Fig. 6H–I’), but often completely absent (Fig. 6G–G’, white hollow arrowheads), and BMP signaling activity was never observed in the ciliated tract of the subpharynx region. Active BMP signaling in the ciliated tract seemed to be localized to the cells at the top rather than at the bottom of the folds (Fig. 6I–I’). It remains unclear if these differences in the detection and localization of pSMAD1/5 signals reflect dynamic changes of BMP signaling in the ciliated tract or they are due to technical limitations of the antibody staining.

In line with the expression of the BMP receptors and pSMAD1/5 immunoreactivity (Fig. 6C-F; H-I’), in situ hybridization analysis also showed the expression of BMP ligand genes in the septal filament. We found *bmp2/4* (Fig. 6J–P) and *bmp5-8* (Fig. 6Q–T) expression in the ciliated tract, curiously, at the bottom of the folds, i.e., in a domain complementary to the areas of pSMAD1/5 activity at the top of the ciliated track folds. Unlike *bmp2/4*, in addition to the ciliated tract expression, *bmp5-8* is also strongly expressed in the reticular tract of the septal filament (Fig. 6Q-S). In agreement with in situ hybridization data, transgenic adult polyps of the *bmp2/4::mCh* reporter line displayed mCherry expression in the mesenterial gastrodermis and at the basis of the ciliated tracts, both in the subpharynx and the gonadal region (Fig. 6M–P,Additional file 1:Fig. S5).

**Fig. 7:**
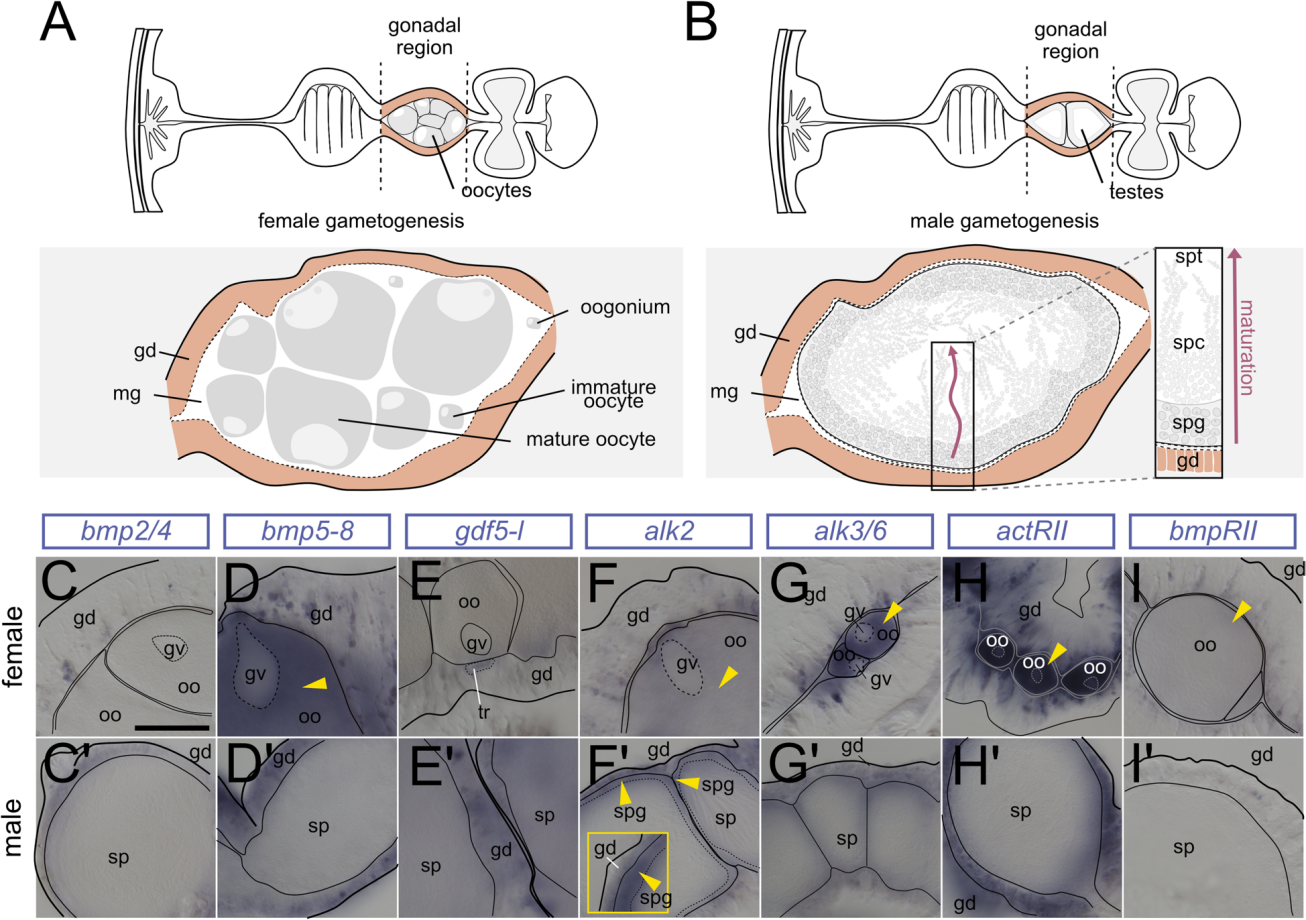
Expression of BMP ligand and receptor genes in maturing female and male gametes. **A,B** Schematic of the mesentery morphology at the level of the gonad as transversal cross-section, gonad region is highlighted in pink. **A** Oogenesis. **B** Spermatogenesis (red arrows indicates the progression of maturation from spermatogonia to sperm). **C–I’** BMP pathway gene expression analysis by in situ hybridization on cross-sections of the female (**C–I**) and male (**C’–I’**) gonadal region. **C–I** Yellow arrowheads indicate detectable transcript in the oocytes (**F’**) *alk2* expression in spermatogonia (yellow arrowheads). All stainings were performed at least two times independently with 3 or more animals imaged. Scale bar 100 µm; mg—mesoglea, gd—gastrodermis, oo—oocyte, gv—germinal vesicle, sp—spermaries, spg—spermatogonia, spc—spermatocytes, spt—spermatids and sperm

In the gonads (Fig. 7A,B), transcripts of BMP ligands *bmp2/4*,* bmp5-8*, and *gdf5-like*, and BMP receptors *alk2*, *alk3/6*, *actrII*, and *bmprII* were detectable in the gastrodermis of the somatic gonad (Fig. 7C–I’), and several of the BMP signaling components were deposited in maturing gametes. In female polyps, in situ hybridization suggested maternal deposition of mRNAs of *bmp5-8*, *alk2*, *alk3/6*, *actrII*, and *bmprII* but not of *bmp2/4* or *gdf5-like* (Fig. 7C–I). This was further supported by the RNA-Seq data from the NvERTx database [43–45], detecting maternal transcripts of *bmp5-8*, *alk2*, *alk3/6*, *actrII*, and *bmprII* in unfertilized *Nematostella* eggs. According to the NvERTx, low *bmp2/4* and *gdf5-like* appear to be also provided maternally, however at much lower levels than *bmp5-8* and undetectable by in situ hybridization (Additional file 1:Fig. S8). In situ hybridization analysis of the male polyps showed expression of *bmp2/4*, *bmp5-8*, *gdf5-l*, *alk2*, *alk3/6*, and *actrII* in the somatic gonad, but no detectable staining of BMP RNAs in the germ cells. In contrast, mRNA of the BMP receptor *alk2* appears to accumulate in the periphery of the spermaries (i.e., in spermatogonia) (Fig. 7C’–I’).

### BMP signaling is absent from vasa-positive germ cells and stem cells

**Fig. 8:**
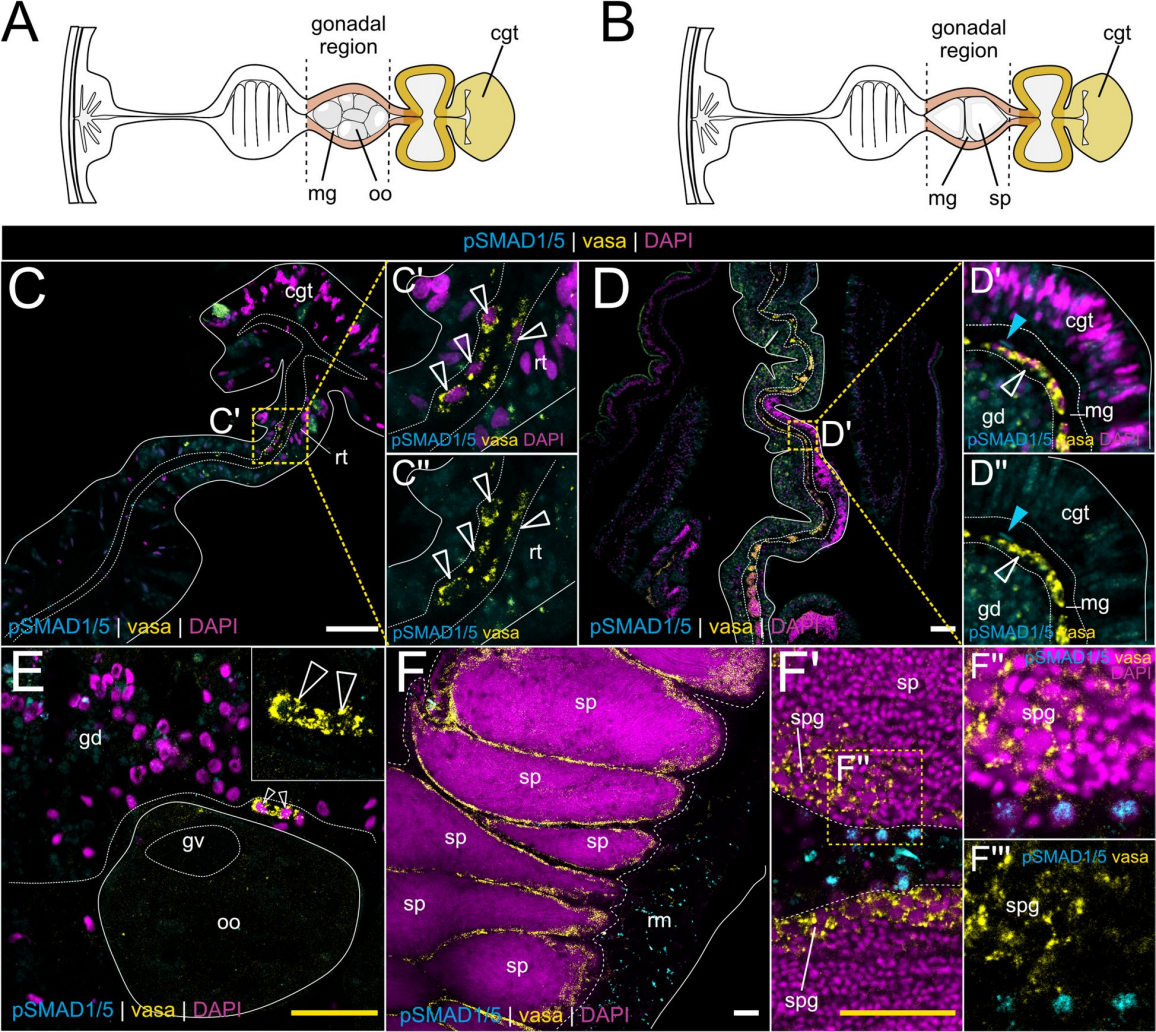
BMP signaling is absent from vasa2-positive germ and stem cells.** A,B** Schematic of the mesentery morphology at the level of the gonad region in female (**A**) and male (**B**) on a transverse section. Gonad highlighted in pink, septal filament highlighted in shades of yellow. **C–F** Immunostaining of pSMAD1/5 (cyan) and vasa (yellow) in different parts of the gonad region. DNA counterstaining is shown in magenta. **C** vasa2-positive basiepithelial cells in the reticular tract are pSMAD1/5-negative. **D** In the gonad region, vasa2-positive basiepithelial cells are pSMAD1/5-negative (white arrowhead). Rarely, pSMAD1/5-positive, vasa2-negative basiepithelial cells are present (blue arrowhead). **E** vasa2-positive putative germ cells and maturing oocytes are pSMAD1/5-negative. **F** vasa2 accumulation in the spermatogonia. **F’–F”’** Sporadic pSMAD1/5-positive cells in the male gonad do not overlap with vasa2 in the spermatogonia. All stainings were performed at least two times independently with 3 or more animals imaged.mg—mesoglea, oo—oocyte, sp—spermaries, cgt—cnidoglandular tract, rt—reticular tract, rmr—retractor muscle region, gd—gastrodermis, gv—germinal vesicle, spg—spermatogonia

Single-cell transcriptomic analyses suggested the upregulation of BMP pathway genes in the previously identified stem cell cluster [18]. While the *Nematostella* stem cell system is still poorly characterized, vasa2 protein was reported to not only mark primordial germ cells, maturing oocytes, and the spermatogonia [46–48] (see Fig. 8A,B) but also a recently described population of putative stem cells in the mesentery contributing to germline and somatic lineages [41]. To determine if BMP signaling is active in any of these populations in the mesentery, we performed immunostaining against pSMAD1/5 in combination with the antibody staining against *Nematostella* vasa2 [48]. In agreement with previous studies, we detected vasa2-positive basiepithelial cells in the reticular and gonad tracts in the subpharyngeal (Fig. 8C–C’) and gonadal region (Fig. 8D–D’), yet there appears to be no overlap with pSMAD1/5 activity (Fig. 8C”, 8D”). In rare instances, we detected pSMAD1/5-positive basiepithelial cells located in the gonad region (Fig. 8D”, blue arrowhead) that were, however, distinct from vasa-positive cells (Fig. 8D”, white hollow arrowhead). In line with what has previously been described by us and others, vasa2 protein was present in maturing oocytes and basiepithelial germ cells in the female gonad, and in the outermost region of the spermaries populated by the spermatogonia [41, 46, 48, 49], yet gametes and putative germ cells displayed no pSMAD1/5 immunoreactivity (Fig. 8E–F”). Occasionally, we observed pSMAD1/5-positive cells that were negative for vasa2 protein in close proximity to the vasa-positive spermatogonia (Fig. 8F”-F”’).

In summary, we did not observe an overlap between vasa2-positive basiepithelial cells and pSMAD1/5-positive basiepithelial cells. Strikingly, BMP signaling is active in basiepithelial cells located in the basal mesentery region. These cells are vasa2-negative; however, it is conceivable that they may represent a progeny of vasa2-positive stem cells and cluster together with the stem cells in the single-cell transcriptomic data.

### BMP signaling in the gonad region is active in female trophonemata and male ciliated plugs

**Fig. 9:**
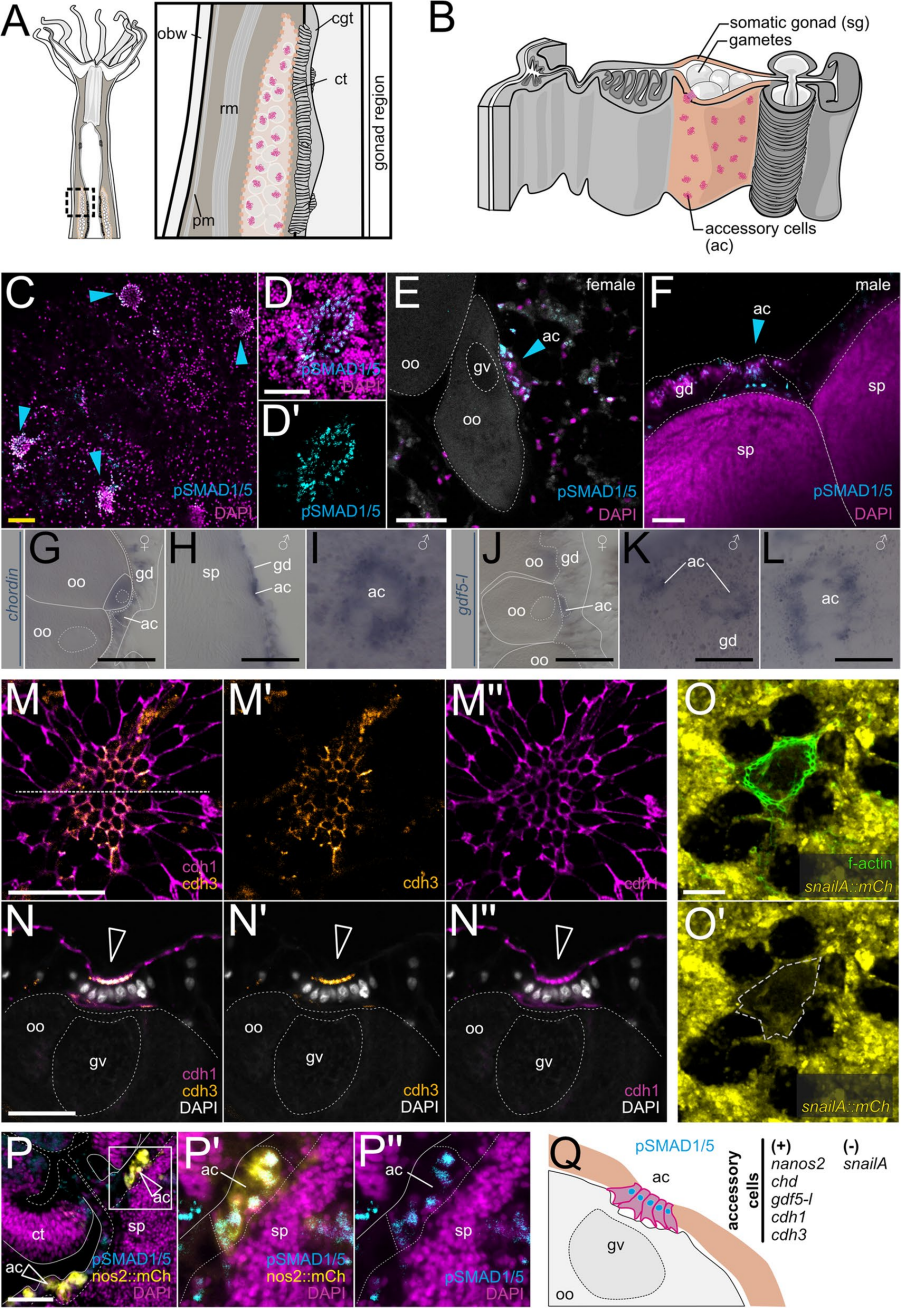
BMP signaling is active in accessory cells in the somatic gonad gastrodermis. **A,B** Schematics of the mesentery morphology in the gonad region in a lateral view (**A**) and in a transverse section (**B**). Gonad highlighted in light pink, accessory cells in magenta. **C–F** pSMAD1/5-positive accessory cells (blue arrowheads) in the gonad gastrodermis are in direct contact with **E** maturing oocytes and **F** spermaries. Expression of **G–I** *chordin* and **J–L** *gdf5-l* in female and male accessory cells*.* **M–N”** cdh1 and cdh3 localization in gonad gastrodermis and accessory cells. cdh1 is present in the apical cell junctions throughout the gastrodermis, while cdh3 is specific to accessory cells (hollow white arrowheads on **N–N”**). **O** f-actin staining shows apical constriction of accessory cells (**O–O’**) *snailA::mCherry* transgenic reporter [50] labels gastrodermal cells but it is excluded from accessory cells. **P–P”** mCherry/pSMAD1/5 double-positive accessory cells in the *nanos2::mCherry* transgenic reporter line [49]. All stainings were performed at least two times independently with 3 or more animals imaged. Scale bars 100 µm (black), 50 µm (yellow), 25 µm (white). ac–accessory cells, sg—somatic gonad, gd—gastrodermis, oo—oocyte, gv—germinal vesicle, sp—spermaries, ct—ciliated tract, obw—outer body wall

Another exception to the low BMP signaling activity of the medial mesentery we discovered in the accessory cells of the somatic gonad. These accessory cells are the trophonema cells specific for the female, and ciliated plug cells specific for the male [49]. They form small islands in the somatic gonad gastrodermis in close proximity to maturing gametes (Fig. 9A,B). In the accessory cells of both, male and female polyps, we detected low levels of pSMAD1/5 activity (Fig. 9C–F). In our in situ hybridization analysis of the somatic gonad, we could not observe detectable levels of BMP receptor expression there; however, trophonema and ciliated plug cells were positive for the BMP ligand *gdf5-l* and the BMP antagonist *chordin* RNA (Fig. 9G-L).

While accessory cells have been addressed in histological and ultrastructure studies [51, 52], they could not be clearly assigned to any cell cluster by single-cell transcriptomic analyses. Since no distinct molecular signatures are known for the trophonemata and ciliated plug cells, we aimed to further analyze these cell populations. Trophonemata and ciliated plugs are apparent due to their tight aggregation and apical constriction of the cells in them compared to the circumjacent gastrodermal epithelium easily discernible in classical cadherin and fibrillar actin stainings (Fig. 9M–O’). *Nematostella* has two classical cadherins: cadherin1 (cdh1) and cadherin3 (cdh3). cdh1 is expressed in the gastrodermis of the mesenteries and in both layers of the body wall, but it is depleted from the epidermis of the tentacles; cdh3 is expressed in the epidermis of the body wall and, especially strongly, in the epidermis of the tentacles, as well as in the pharynx-derived tissue including the septal filament [53] (Additional file 1:Fig. S9). Surprisingly, in the otherwise cdh1-positive/cdh3-negative epithelium of the gonad, cdh3 was clearly detectable in the contacts between the apically constricted cells of the trophonemata and ciliated plugs (Fig. 9M–N”). Side views of the trophonema (Fig. 9N–N”) showed colocalization of cdh3 and cdh1 at the apical side of the cells facing the gastric cavity, while only cdh1 was present at the basal trophonema side facing the animal pole of the oocyte, where the germinal vesicle is located (Fig. 9N–N”). In line with the differential expression of cadherins, we observed the downregulation of mCherry expression in the trophonemata in the *snailA::mCherry* reporter line compared to the rest of the gonad gastrodermis (Fig. 9O–O’), suggesting the repression of cdh3 in the mesentery gastrodermis by SnailA, similar to the situation reported in the embryo [53]. Moreover, recently, trophocytes have been shown to express the RNA-binding protein Nanos2 [49], which we have recently identified as a direct target of pSMAD1/5 during early development [27]. In line with that, we found pSMAD1/5 activity overlapping with the mCherry expression in the *nanos2::mCherry* transgenic reporter line (Fig. 9P–P”).

### The medusozoan species *Aurelia coerulea* and *Tripedalia cystophora* display broad BMP signaling activity across different body regions

**Fig. 10:**
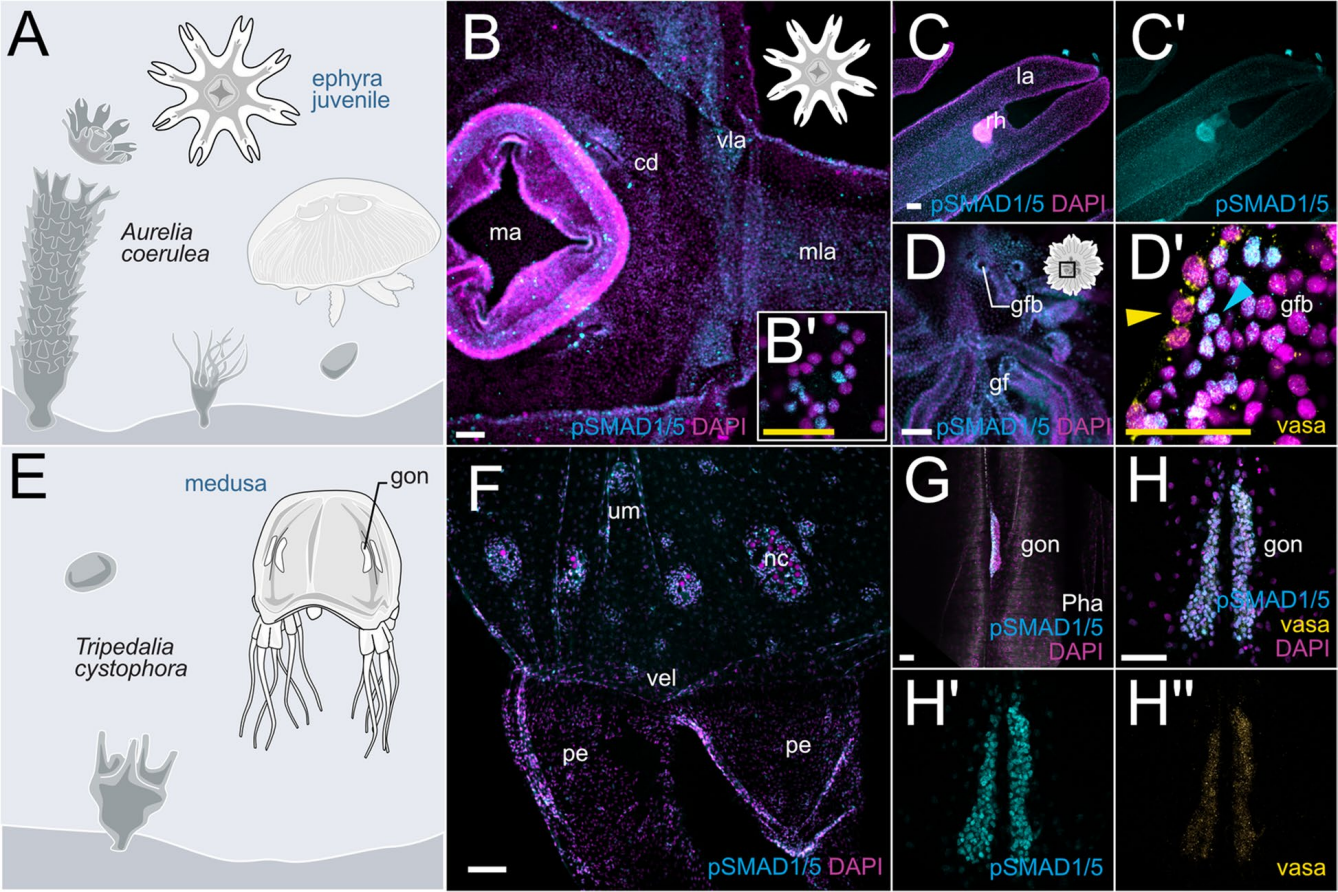
BMP signaling is broadly active in medusozoans, the scyphozoan jellyfish *Aurelia* and the cubozoan jellyfish *Tripedalia*. **A** Scheme of the life cycle of *Aurelia coerulea*. **B–D’** pSMAD1/5-positive nuclei in the ephyra (**B–B’**) central disc (cd), strong staining in the manubrium (ma) is likely unspecific (non-nuclear), **C–C’** lappet and rhopalia. **D–D’** vasa2-staining in the region of the gastric filaments (yellow arrowhead) shows no overlap with pSMAD1/5-positive nuclei (blue arrowhead). **E** Scheme of the life cycle of *Tripedalia cystophora*. **F** Broad pSMAD1/5 activity in the umbrella and velarium. **G** *Tripedalia* gonad is pSMAD1/5-positive. **H–H”** Germ cells in the *Tripedalia* gonad are pSMAD1/5- and vasa-positive. All stainings were performed two times independently with 4 animals imaged. Scale bar 50 µm (white) and 25 µm (**B’**, yellow). ma—manubrium, cd—central disc, vla—velar lappet, mla—marginal lappet, gf—gastric filament, gfb—gastric filament base, um—umbrella, nc—nematocyst wart, vel—velarium, pe—pedalium, gon—gonad

The expression of several BMP components, but mostly of BMP ligands, has been previously analyzed in several members of Medusozoa, including *Hydra*, *Clytia*, *Podocoryne*, and *Aurelia* [30–32, 54], yet nothing is known about the activity and localization of BMP signaling in jellyfish and hydroids. Therefore, we aimed to test the cross-reactivity of the pSMAD1/5 antibody in multiple medusozoan representatives. We failed to detect specific anti-pSMAD1/5 staining in any of the assayed hydrozoan species including *Clytia hemisphaerica*,* Hydra vulgaris*, *Hydractinia sp.*, and *Ectopleura sp.* and a scyphozoan species, *Sanderia malayensis* (Additional file 1:Fig. S10). In contrast, we successfully detected BMP signaling by pSMAD1/5 staining in the scyphozoan jellyfish *Aurelia coerulea* and the cubozoan jellyfish *Tripedalia cystophora*. Both species feature a triphasic life cycle common for most Medusozoa, with a free-swimming larva, a sessile polyp, and a free-swimming medusa stage (Fig. 10A, E).

In *Aurelia*, we focused on the developmental stages that were most accessible for whole-mount immunofluorescence staining: the polyp stage and early juvenile medusa stages (ephyra and metaephyra). *Aurelia* polyp (scyphistoma) undergoes serial transverse fission and releases multiple genetically identical juvenile jellyfish (ephyrae), which develop into the metaephyra and, subsequently, into adult medusae (Fig. 10A). No active BMP signaling was observed in the unsegmented *Aurelia* polyp (Additional file 1:Fig. S10), while at the ephyra stage, BMP signaling activity was present across different portions of the body. Here, mixed populations of pSMAD1/5-positive and -negative cells were found in the epithelium of the central disc, the lappets, and the sensory organs called rhopalia (Fig. 10B,C). In contrast, the manubrium appeared to be free of BMP signaling, although we observed a strong background signal and unspecific (non-nuclear) staining of putative muco-glandular cells (Fig. 10B), similar to the unspecific staining observed in the *Nematostella* pharynx and septal filament. At the metaephyra stage, which is characterized by the stepwise fusion of the individual lappets into a bell-shaped umbrella, BMP signaling was observed in similar areas as in the ephyra. In addition, pSMAD1/5-positive cells were found at the base of the gastric filaments (Fig. 10D), which are located in four distinct gastric pouches around the manubrium. In contrast, gastric filaments, which are very rich in gland cells, showed only unspecific (non-nuclear) staining. Later during development, the gastric pouches also contain four horseshoe-shaped gonads sitting underneath the gastric filaments. Co-immunostaining with anti-pSMAD1/5 and anti-*Nematostella* vasa2 antibody revealed vasa-positive cells with a typical perinuclear vasa signal at the base of the gastric filaments that were distinct from the pSMAD1/5-positive cells located immediately next to the vasa expressing cells—similar to the situation in *Nematostella* (Fig. 10D’).

In *Tripedalia*, antibody staining revealed a broad pSMAD1/5 activity in juvenile and adult jellyfish. Mixed populations of pSMAD1/5-positive and -negative cells were detectable in the umbrella, velarium, ring nerve, parts of the nematocyst warts, and rhopalia of the jellyfish, while the activity was absent from the manubrium, pedalia, and tentacles (Fig. 10F). In sexually mature females, four pairs of wing-shaped gonads are located on the inside of the medusa bell next to the radial canals of the gastrovascular system. Strikingly, germ cells in the female gonad of *Tripedalia* were pSMAD1/5-positive (Fig. 10G–H’), and co-staining with anti-*Nematostella* vasa2 antibody showed an overlap with the accumulation of vasa protein (Fig. 10H–H”), which was never observed for germ cells in *Nematostella.*

## Discussion

### Diversity of BMP signaling domains in Cnidaria indicates a variety of functions

To date, most of our knowledge on BMP signaling in Cnidaria comes from expression-based studies in Medusozoa, as well as expression-based and functional studies in anthozoan embryos [26–32, 36–39, 55]. Research in anthozoans, however, only addressed the role of BMP signaling in directive axis establishment and patterning. In this work, we aimed to expand this understanding by investigating the activity of BMP signaling unrelated to axial patterning in the adult polyp of the anthozoan *Nematostella*, and two radially symmetric medusozoans, the juvenile ephyra of the scyphozoan *Aurelia* and the cubozoan jellyfish *Tripedalia*, utilizing an antibody against the BMP effector pSMAD1/5 [26–28]. In all three species, BMP signaling was active in distinct cell populations in different locations, suggesting that BMP signaling must be involved in the regulation of multiple processes.

**Fig. 11:**
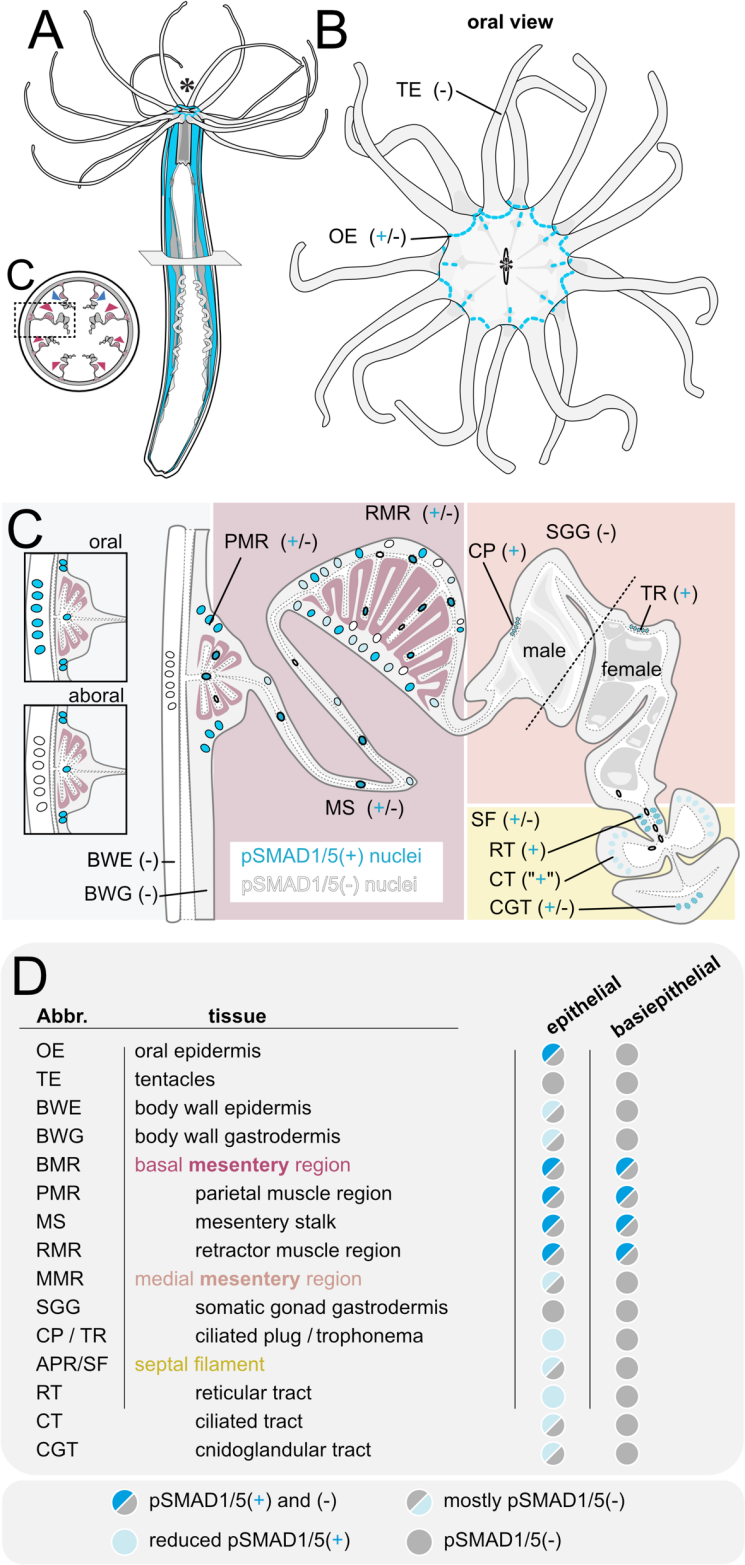
Summary of BMP signaling domains in the adult *Nematostella* polyp

We centered our analysis on *Nematostella* and systematically dissected BMP activity domains in this bilaterally symmetric model sea anemone. We showed that BMP signaling was strongest in distinct areas of the epidermis of the oral region, as well as in specific cell populations in the gastrodermis of the mesenteric folds (Fig. 11A–D). In the mesentery, pronounced BMP activity was detected in the basally located neuro-muscular region, while it appeared reduced, although not absent, in the more apical parts including the gonad region. In line with that, our analysis of the published single-cell transcriptomic data indicates broad expression of the BMP pathway genes [18]. The diversification of BMP activity in multiple specialized cell types may already start during larval development, after patterning of the directive axis is completed, as indicated by our previous anti-pSMAD1/5 ChIP analysis. We identified direct BMP signaling target genes at late gastrula, when the axis patterning gradient of BMP signaling activity has just formed, and in 4d planula, when the axis is already patterned, and the pSMAD1/5 gradient is no longer present. While at both stages, many BMP targets are developmental regulators involved in the patterning of the directive axis, in 4d planula we observed an increasing number of targets with other putative functions such as metabolism, extracellular matrix dynamics, or neurogenesis [27]. In the future, it will be important to follow the dynamics of BMP signaling between 4d planula and adult and figure out how adult BMP signaling domains appear.

In medusozoans, BMP activity and the expression of BMP signaling components appear to be similarly versatile. Our study revealed pSMAD1/5 being broadly active in the epithelium of the central disc, radial canal and lappets, as well as in the rhopalia of *Aurelia* and *Tripedalia*. In line with that, earlier studies in the ephyra of *Aurelia* and medusa of *Clytia* and *Podocoryne* detected *bmp5/8* expression in the radial canal and sensory structures (rhopalia) [30]. In *Hydra*,* HySmad1* mRNA is absent from the tentacles and the peduncle but broadly detectable along the entire body column, where it is expressed in a variety of cell types including epithelial cells, neurons, gland cells, interstitial stem cells, and endodermal epithelium, as determined by northern blot on mRNA from fractionated cells [54]. In contrast, a *bmp2/4*-like gene and three copies of *bmp5-8* are expressed at the tentacle base, as well as in the body column [32, 55–59].

Together, our data on BMP activity domains in three cnidarian representatives, combined with existing cnidarian expression data, indicate that BMP signaling may be relevant in at least three different contexts, besides axial patterning: (1) in tentacle formation, (2) in the neuro-muscular region, and (3) in the reproductive region.

### Potential role of BMP signaling in tentacle formation

Among the most pronounced domains of BMP signaling in *Nematostella* polyp was the pSMAD1/5 activity between the tentacles (Fig. 11B, D). A similar pattern of BMP activity can be observed during postembryonic development, when BMP signaling forms a cross-like activity domain in the oral epidermis [27] (Additional file 1:Fig. S3), with four pSMAD1/5-free territories at the localization of the four primary tentacle buds. Starting from the 4d planula, pSMAD1/5 activity is also accompanied by the expression of several BMP components and BMP target genes in the tentacle area. The pSMAD1/5-negative tentacle buds express genes encoding BMP2/4, BMP5-8 (Additional file 1:Fig. S3B-C), BMP antagonists Noggin and Gremlin [37], a metalloprotease Tld [38] and a BMP and BMP receptor binding molecule RGM [28] (Additional file 1:Fig. S3D-D’). In contrast, the pSMAD1/5-positive intertentacular area expresses genes encoding a BMP-binding protein CV2 [60] (Additional file 1:Fig. S3E-E’) and another BMP ligand, ADMP (Additional file 1:Fig. S3F-F’). Single-cell transcriptomic data indicates that *admp* is the only BMP gene, whose expression persists in the epidermis of the adult polyp making it the prime candidate for signaling in the intertentacular domain at this stage.

Intertentacular pSMAD1/5 suggested potential role of BMP signaling in preventing tentacle formation, and this notion is supported by the phenotype observed in *bmp2/4* mutants we generated using CRISPR/Cas9-mediated gene editing. While most injected F0 embryos died after developing a “worm phenotype” described previously for the *bmp2/4* morphants [28], some embryos survived until adulthood. Many of these adults formed large bubble-like structures on the sides of their bodies. Genotyping showed that in the F0 animals, the bubbles contained *bmp2/4* mutant cells, and once we obtained heterozygous *bmp2/4*^±^ F1, these animals also started sporadically developing bubbles (Additional file 1:Fig. S11B-C). Morphological analysis confirmed the presence of epidermal muscles and spirocytes in the bubble tissue (Additional file 1:Fig. S11D-E’), both of which are unique features of a normal *Nematostella* tentacle. Moreover, qPCR analyses showed that *cdh3* (a cadherin specifically upregulated in the tentacles [53]) and *nem64* (a tentacle muscle-specific bHLH transcription factor [34]) are upregulated in the bubble of the F0 mutants 7.7-fold and 7.6-fold, respectively, while *cdh1* (a cadherin specifically upregulated in the body wall and downregulated in the tentacle [53]) is 2.6-fold downregulated (Additional file 1:Fig. S11F-G). Thus, the bubbles developing in the mosaic and heterozygous *bmp2/4* mutants appear to be products of an ectopically and improperly activated tentacle formation program.

Expression data suggest the association of BMPs with the tentacles in several Medusozoa. In the polyps of the hydrozoans *Hydra*, *Podocoryne*, and *Clytia*, as well as in the polyp of the scyphozoan *Aurelia*, BMP ligand *bmp5-8* is expressed in developing tentacle buds [30–32]. In the case of *Hydra*, several other BMPs, including *bmp2/4-like* and *bmp5-8c*, also show increased expression at the tentacle base in a homeostatic polyp, and all *bmp* genes react dynamically to budding and/or head regeneration [32, 58].

Taken together, we see evidence for the involvement of BMP signaling in regulating patterning and morphogenesis of the tentacle domain in *Nematostella* as well as in several medusozoans. However, the exact role of BMP signaling in this and the way BMP signaling acts in concert with other pathways, e.g., with FGF and Hedgehog signaling in *Nematostella* [46, 61], remain to be discovered.

### Potential role of BMP signaling in neurons, muscles, and cnidocytes

Our analysis of the single-cell transcriptomic data shows upregulation of BMP pathway genes in *Nematostella* neurons, muscles, and cnidocytes. Neurons and cnidocytes are generated by a common pool of SoxB2-positive multipotent progenitors during embryonic, larval, and post-larval development [35, 62], while muscles originate from other cells. Our data in the adult polyp demonstrate that domains of pronounced BMP signaling activity coincide with the basal part of the mesentery (Fig. 11C,D) and include two of the three neuron-rich areas of the gastrodermis: the longitudinal nerve tracts at base of the mesentery located next to the parietal muscle, and the intramesenterial neurons, some of which are embedded into the retractor muscle, but not the neurons of the body wall and the circular muscles.

ChIP-seq with an anti-pSMAD1/5 antibody on late gastrula and 4d planula of *Nematostella* revealed multiple genes as BMP targets including *arp6*, *ashB*,* atoh7*, *hmx3*, *isl*, *irx*, *lmx*, *soxC, rgm*, *ephrinB*, and *netrin*, which are associated with neural function in vertebrates and invertebrates [27]. In contrast, we do not find many muscle-related genes among the direct BMP signaling targets, which suggests mesenterial neurons rather than parietal or retractor muscle cells as more likely candidates for being pSMAD1/5-positive. Despite being vital during formation and patterning of the centralized nervous system in many Bilateria, the roles of BMP signaling in the formation of the diffuse nerve net common in Cnidaria are largely unknown. In *Nematostella*, early neurogenesis is considered to be independent of BMP signaling [63, 64], with pSMAD1/5 becoming detectable in the oral domain during gastrulation, which is after the onset of the neural gene expression at the blastula stage and in a different location in comparison to where the first RF-amide-positive and elav-positive neurons are found in the gastrula [27, 62, 65]. In line with that, knockdown and upregulation of BMP signaling showed no effects on early neurogenesis; however, an anti- as well as a pro-neurogenic potency of BMP signaling has been observed during oral nerve net formation in the planula larvae [64].

While some neurons appear to be pSMAD1/5-positive, the situation with cnidocytes is different. Transcripts of the BMP pathway genes are expressed in mature cnidocytes in the single-cell data (Fig. 1E); however, we did not observe nuclear pSMAD1/5 in any stinging cell subtype, besides the unspecific, non-nuclear signal in spirocytes (Additional file 1:Fig. S4A–A’). Notably, the cnidocyte-specific expression of some of the BMP components may be shared with other cnidarians, given the fact that *HySmad1* could be detected in mature nematocytes of the gastric region in *Hydra* [54]. Co-staining of pSMAD1/5 in different existing transgenic reporter lines [35, 62, 66] or together with antibody staining for neuronal, muscle, and nematocyte markers will help elucidate the role of BMP signals in the development, regional patterning, and maintenance of neurons, cnidocytes, and muscles in *Nematostella* and, eventually, other cnidarians.

### Potential role of BMP signaling in the reproductive region

While we have a comprehensive understanding of the stem cell system in hydrozoan species [57, 67–69], most efforts to characterize stem cells in non-hydrozoan cnidarians remained unsuccessful. Ultrastructural studies in Anthozoa and Scyphozoa revealed extraepithelial, amoeboid cells, which were proposed to be stem cells [42, 70] but lack further characterization. A recent study in *Nematostella* provided evidence for a *vasa2*/*piwi1* double-positive basiepithelial putative stem cell population in the mesentery in the basal portion of the septal filament [41]. As these adult stem cells contribute to vasa2-positive primordial germ cells (PGCs) as well as to the soxB2-positive neuronal progenitors, they were proposed to be homologous to the interstitial stem cells (i-cells) of Hydrozoa [41].

Our analysis of *Nematostella* scRNA-seq data indicated the conspicuous upregulation of BMP pathway members in the previously identified stem cell cluster [18]. In accordance with the observations by Miramon et al. [41], we detected vasa2-positive basiepithelial cells in the septal filament (Fig. 8C,D); however, these cells were pSMAD1/5-negative. Instead, we found an upregulated expression of BMP ligands *bmp2/4* and *bmp5-*8 as well as pSMAD1/5-positive cells in the epithelial parts of the septal filament, where vasa-positive basiepithelial cells concentrate. Curiously, pSMAD1/5 is detectable in vasa2-negative basiepithelial cells located in the neuromuscular domain of the mesentery. Considering that single-cell transcriptomic data indicated the upregulation of BMP pathway genes in putative stem cells, these findings suggest that either *vasa2*/*piwi1*-positive adult stem cells express BMP components but do not receive BMP signals or that there are other, *vasa*-negative stem cell populations that may be pSMAD1/5-positive.

Curiously, expression data of BMP components in Hydrozoa also implies possible involvement of BMP signaling in interstitial stem cell and germ cell development. Hydrozoan i-cells constitute a pool of pluri- or multipotent stem cells that contributes to somatic cells and the germline [67, 69, 71–73]. In *Hydra*, Northern hybridization of *smad1/5* indicated that it is upregulated in i-cells [54], while transcriptomic analysis showed the upregulation of the BMP receptor *actr1* in multipotent interstitial cells and *smad1/5* and BMP antagonist *dand* in germline stem cells [74]. In *Hydractinia*, the expression of a putative *bmp receptor* has been detected in developing oocytes and the male gonophore gastrodermis [33]. *bmp5-8* has been also found to be differentially regulated in female and male *tfap2*-positive germ cells in *Hydractinia* [75]. Another TGFβ molecule, *gonadless* (*Gls*), which may or may not be a BMP, is expressed in *tfap2*-positive germ cells, and *gls* knockout does not affect the germ cells but results in the absence of the sexual zooids carrying gonads in *Hydractinia* [76].

All these observations in hydrozoans suggest a role of BMP signaling in gonado- or gametogenesis. This role appears to be highly conserved across Bilateria [10], and our analysis suggests that BMP signaling related to reproductive processes might be conserved in *Nematostella*, *Tripedalia*, and *Aurelia*. Single-cell transcriptomic data from the adult *Nematostella* polyp suggest that BMP genes are not expressed in primordial germ cell (PGC) clusters, and we never observed pSMAD1/5 activity in the vasa2-positive stem and germ cell lineage, including maturing gametes (Fig. 8C,D); however, RNA of several BMP signaling components was detected in the developing oocytes (*bmp5-8* and *alk6*) and spermatogonia (*alk2*). Similar to *Nematostella*, the juvenile *Aurelia* metaephyra displayed pSMAD1/5-positive cells in proximity to the developing reproductive region in the gastric pouches, but without showing an overlap with vasa-positive cells. Notably different is the situation in *Tripedalia*, where BMP signaling was specifically active in vasa-positive germline cells in the gonad.

One interesting cell population in this context is the cnidarian-specific accessory cells described in Anthozoa and Scyphozoa [49, 51, 52, 77–82]. *Nematostella* accessory cells, termed trophocytes in the female and ciliated plug cells in the male polyp, are located in the somatic gonad in a close contact with maturing oocytes and the spermatogonia (Fig. 9C–F). Little is known about their function; however, unlike previously suggested, trophonemata appear not to be involved in nutritional transport [79] but may play a role during germ cell development [49] or, given the differential cadherin expression we found, serve as release points for the gametes. While the somatic gonad gastrodermis is generally pSMAD1/5-negative, we detected active BMP signaling in accessory cells of the female and male polyp, also expressing the BMP ligand *gdf5-l* and the BMP antagonist *chordin*. Accessory cells display an upregulation of RNA-binding zinc finger molecule *nanos2*, a gene expressed in the piwi1/vasa2/nanos2-positive multipotent stem cell population and in a broad progeny of the neuroglandular lineage [49]. Furthermore, *nanos2*^−/−^ knockouts not only lack accessory cells but also fail to establish the germline [49]. While we cannot comment on the regulatory relationship of BMP signaling and *nanos2* expression in the adult polyp besides them being active together in accessory cells, *nanos2* is a direct target gene of pSMAD1/5 during larval development [27].

## Conclusions

In this study, we provide a whole-body atlas of BMP signaling activity in the adult bilaterally symmetric cnidarian model *Nematostella* and a brief overview of BMP signaling activity in two radially symmetric cnidarian models, *Aurelia* and *Tripedalia*. Currently, we are unable to state with certainty which cell types/states from single-cell transcriptomic data are the ones receiving BMP signals in the adult *Nematostella* polyp; however, in the future, as more and more adult cell states become “de-orphanized,” this question will no doubt be resolved. The same approach will then have to be expanded to our two medusozoan models, *Aurelia* and *Tripedalia*. Our analyses show that BMP signaling is involved in a variety of processes beyond axial patterning, which explains the presence of the BMP signaling components in non-bilateral Cnidaria. Among these processes, the role of BMP signaling in regulating tentacle formation, neuronal differentiation, and gameto- or gonadogenesis clearly stand out and will form focal points of the future studies.

## Methods

### Animal culture

*Nematostella vectensis* adult polyps, separated by sexes, were maintained in the dark at 18 °C in 16 ppm artificial seawater (*Nematostella* medium, NM) and spawned as described before [83, 84]. *Aurelia* polyps (scyphistomae) were cultured in 35‰ ASW (artificial sea water) in small Petri dishes at 20 °C. Strobilation was induced by temperature change (polyps were transferred to ~ 15 °C for several hours and then again kept at 20°). *Clytia* polyp colonies and jellyfish were maintained in 35‰ ASW in kreisel tanks. *Tripedalia* jellyfish and *Sanderia* ephyra were obtained from the Tiergarten Schönbrunn, Vienna, *Hydractinia* from the Haus des Meeres, Vienna, and directly processed for immunostaining. *Ectopleura* polyps were collected in Grado, Italy, and processed for immunostaining in Vienna.

### *Nematostella bmp2/4* mutagenesis

To generate *bmp2/4* mutants, the genomic sequence encoding for the N-terminus of the mature BMP2/4 ligand was targeted using the ALT-R CRISPR-Cas9 system (IDT). crRNAs targeting two (overlapping) sequences were ordered: AAACGGAGCCTGCGGTCCGGCGG and AGAAAACGGAGCCTGCGGTCCGG, and both proved to work efficiently. Genotyping was performed using the primers GCAGACAACGATGGGATTGACGCTAGTG and GTATTGCCCGTTCTAATCATCCTTGAAGGC.

Quantitative PCR on wild type and bubble tissue cDNA was performed with the following primers: for *cadherin3* (NV2.4928 or NVE8896), cdh3_qF GCTACATCTTCCGTCTCGGG and cdh3_qR TAGTTGCCCTCACGGTTCAC; for *cadherin1* (NV2.4715 or NVE818), cdh1_qF AGTCGTGAATGCCCCAGAAG and cdh1_qR ACACGCAAATGCTGTCCCTA; for *nem64* (NV2.10867 or NVE19721), nem64_qF TGACGCCTTCTCCTGAACCA, and nem64_qR CGTTCTCGTGCGTTCGCTAA. Primers against GAPDH were used as normalization control. Boxplots were created using the boxplot web tool (http://shiny.chemgrid.org/boxplotr/).

### Fixation for immunohistochemistry

Adult *Nematostella* were starved for up to 3 days and left in 0.1 M MgCl_2_ in NM until fully relaxed (5–15 min). For fixation, polyps were transferred to a new dish containing ice-cold fixation solution 1 (3.7% Formaldehyde in PBS). For antibody staining of pSMAD1/5, fixation solution 1 (3.7% Formaldehyde, 1 × PBS, 0.2% Triton-X100) was used. For antibody staining of pSMAD1/5, vasa2, cdh1, and cdh3, fixation solution 2 (3.7% Formaldehyde, 0.4% glyoxal, 0.1% MeOH, 1 × PBS, 0.2% Triton-X100) was used. To ensure consistent penetration, the fixation solution was injected slowly through the mouth opening, using a 1-ml syringe and a Sterican blunt needle with a bent tip. Polyps were transferred to a new tube with fresh, ice-cold fixation solution and fixed for 60 min under rotation at 4 °C. After 20–30 min fixation time, samples were transferred to a dish with fresh fixation solution for further dissection. For vibratome sectioning, samples were cut into ~ 5-mm pieces transversely through the body column. For whole mount imaging, the body column was opened longitudinally, and mesenteries were extracted from the body wall. After dissection, the fixation was continued under rotation at 4 °C. The fixative was removed in 3 washes with 1 × PBS / 0.2% Triton-X100. To remove pigments, samples were repeatedly washed with 100% methanol until the liquid remained clear. Samples were washed 3 × with 1xPBS, 0.2% Triton-X100, and directly further processed. For anti-pSMAD1/5 staining, samples were incubated in Bloxall™Blocking Solution (Vector Laboratories) before the antibody blocking step (see “Immunohistochemistry”) to reduce unspecific signal. Samples were incubated with Bloxall for 10–30 min at room temperature, followed by 3 × washes with 1 × PBS.

Ephyra of *Aurelia* and *Sanderia*, polyps of *Aurelia*,* Hydractinia*,* Ectopleura*, and jellyfish of *Clytia* and *Tripedalia* were relaxed in 0.1 M MgCl_2_. *Hydra* polyps were relaxed in 2% urethane for 2 min. Samples were fixed using 3.7% Formaldehyde in 1 × PBS and 0.2% Triton-X100 for 90 min (except *Tripedalia* with 60 min of fixation) at 4 °C under rotation. After fixation, samples were washed 3 times with 1 × PBS / 0.2% Triton-X100.

### Fixation for in situ hybridization

Sample preparation for in situ hybridization of mesentery pieces or pieces of the body column was carried out as described before (see “Fixation for immunohistochemistry”). Sample preparation and fixation was performed at room temperature using fixation solution 1 (3.7% FA, 1 × PBS, 0.2% Triton-X100), fixation was carried out on a rotator for 1 h at room temperature. ISH samples were stored in 100% MeOH at − 20 °C.

### Immunohistochemistry

The same antibody staining protocol was used for all cnidarian species. Samples were blocked in Blocking solution I (1% BSA, 5% sheep serum, 1 × PBS, 0.2% Triton-X100, 20% DMSO) for at least 2 h at room temperature. In parallel, the primary antibodies were pre-absorbed in Blocking solution II (1% BSA, 5% sheep serum, 1 × PBS, 0.2% Triton-X100, 0.1% DMSO). Primary antibody incubation of the sample was carried out overnight at 4 °C with the following primary antibody dilutions: rabbit anti-pSMAD1/5/9 1:200 (mAb #13,820, Cell signaling, [26–28], mouse anti-vasa2 1:1000 [48], rabbit anti-cadherin1 (1:500) and mouse anti-cadherin3 (1:1000) [53], mouse anti-Tyrosinated Tubulin (TUB-1A2, T9028 Sigma, 1:1000). Samples were washed 10 times with 1 × PBS / 0.2% Triton-X100, blocked in Blocking solution II for 1 h at room temperature and incubated with the secondary antibodies 1:1000 (goat α-rabbit IgG-Alexa568 (Invitrogen A11008), goat α-rabbit IgG-Alexa633 (Invitrogen A21070), goat α-mouse IgG-Alexa488 (Invitrogen A11001), goat α-mouse IgG-Alexa568 (Invitrogen A11004) and DAPI, 1:1000) in Blocking solution II at room temperature for 2 h or at 4 °C overnight. After 10 washes in 1 × PBS / 0.2% Triton-X100, they were either infiltrated with and mounted in VECTASHIELD® Antifade Mounting Medium (H-1000–10, VectorLabs) or further processed for vibratome sectioning.

### Vibratome sectioning

Vibratome sectioning was performed after antibody staining or in situ hybridization. Embedding medium (10% gelatin in PBS) was dissolved at 50 °C and stored at − 20 °C in 2 ml tubes. Before use, the embedding medium was warmed at 37 °C until liquified and pieces of the body column were left for infiltration until settling down to the bottom of the tube. Samples in the embedding medium were poured into mounting molds (cut 1-ml pipette tip), pieces were orientated, and molds were transferred to the fridge for hardening for 30 min. Hardened gelatine blocks were fixed in 4% Formaldehyde in PBS at 4 °C overnight. Samples were washed 3 × with 1 × PBS and sectioned at a Leica VT1200 vibratome (speed = 0.5, amplitude = 0.7 and auto feed thickness = 50–100 μm). Sectioned in situ hybridization samples were infiltrated with and mounted in glycerol, immunohystochemistry samples in VECTASHIELD® Antifade Mounting Medium (H-1000–10, VectorLabs).

### In situ hybridization

Urea-based in situ hybridization on adult tissues was carried out as described previously [79], with minor changes. Pieces of *Nematostella* body column or of individual mesenteries were dissected and fixed for 1 h at room temperature (as described in Fixation for immunohistochemistry and Fixation for in situ hybridization). Anti-Digoxigenin-AP (anti-DIG/AP) Fab fragments (Roche) were diluted 1:4000 in 0.5% blocking reagent (Roche) in 1 × Blocking buffer solution (Roche).

### Single-cell transcriptomic analysis

*Nematostella* gene IDs of BMP signaling pathway molecules were obtained from the Nv2 genome browser (https://simrbase.stowers.org/starletseaanemone): NV2.19847 (*bmp2/4*), NV2.6269 (*bmp5-8*), NV2.6489 (*gdf5-l*), NV2.2978 (*admp*), NV2.2977 (*smad1/5*), NV2.10628 (*smad4*), NV2.15308 (*smad4-l*), NV2.13963 (*smad6*), NV2.11643 (*alk2*), NV2.13851 (*alk3/6*), NV2.13010 (*bmprII*), NV2.12106 (*actrII*), NV2.24312 (*rgm*), NV2.14185 (*chordin*), NV2.122 (*gremlinA*), NV2.12016 (*gremlinB*), NV2.23832 (*follistatin*), NV2.12730 (*cv2*), NV2.7393 (*noggin2*), NV2.7394 (*noggin1*). Single-cell transcriptomic analysis of BMP pathway genes was performed using previously published data by Cole et al. [18] (https://github.com/technau). The R-package Seurat vs4 [85] was used to process the count matrices to include adult wild type libraries. The full dataset from Cole et al. [18] was partitioned into samples derived only from adult tissues, and all clusters with less than 10 remaining cells were dropped. Expression of genes of interest across the available clustering was examined using the Seurat::DotPlot function, holding maximum dot size fixed at 100%. R scripts used for the analysis are available on GitHub (https://github.com/technau/Nv2_Atlas, https://github.com/technau/BMPatlas/releases/tag/Vs.BioRxiv) and Zenodo (10.5281/zenodo.14765821).

### Protein IDs and phylogenetic analyses

For the phylogenetic analyses, *Nematostella s*equences (see “Single-cell transcriptomic analysis”) were translated using the expasy webtool (https://web.expasy.org/translate/). Sequences of human BMP receptors and cv2 sequences of different species were obtained from Uniprot: P37023 (ACVRL1), Q04771 (ACVR1), P36894 (BMPR1A), P36896 (ACVR1B), P36897 (TGFR1), Q8NER5 (ACVR1C), O00238 (BMPR1B), P37173 (TGFR2), Q13873 (BMPR2), P27037 (ACVR2A), Q13705 (ACVR2B); A0A8J0VHU6 (BMPER_XENLA), Q8CJ69 (BMPER_MUSMU), Q8N8U9 (BMPER_HOMSA), Q9W2H2 (BMPER_DROME), Q9W494 (BMPER_ DROME). *Oscarella carmela* Chordin model (m.95558) was obtained by BLAST at https://compagen.unit.oist.jp/. *Trichoplax adherens* Chordin-like EST (TriadT52199) was retrieved by BLAST from https://metazoa.ensembl.org/Trichoplax_adhaerens/. Oyster Chordin protein sequence CGI_10022848 was taken from [86], and *Aurelia* Chordin sequence was obtained by BLAST from the published transcriptome [87]. Other Chordin and Chordin-like sequences used in the tree were obtained by NCBI BLAST and are as follows: XP_001625161.2, NP_001081778.1, NP_001259576.1, NP_034023.1, XP_009058760.1, AMY99568.1, AMY99573.1, NP_001036036.1, AAC41250.1, AAI62594.1, XP_019630752.1, NP_001079355.1.

## Supplementary Information

Additional file 1. Fig. S1 – Phylogenetic analyses of *Nematostella* TGFβ receptors and CV2. Maximum likelihood phylogenies were constructed using IQ-tree [88], the bootstrap values are indicated at the nodes. Protein IDs used in the tree are listed in the Methods. Additional File 1: Fig. S2 – Dot plot showing the expression of BMP pathway genes in single-cell transcriptomic data of adult *Nematostella* polyp tissues with fine clustering presenting 91 transcriptomic cell states (36). Percent Expressed – dot size indicates the percentage of cells in a cluster expressing the gene of interest, Average Expression – color indicates averaged expression value. Additional File 1: Fig. S3 – BMP signaling activity and expression of BMP signaling components in the head region. (A-A’) pSMAD1/5 activity between the forming tentacles and around the mouth of the 4d planula. (B) *bmp2/4* expression in the oral gastrodermis and in the aboral epidermis of the planula; faint expression is discernable in the tentacle buds, (C) *bmp5-8* is also expressed in the gastrodermis, tentacle buds and aboral epidermis, but appears stronger than *bmp2/4*. (D-D’) *rgm* is strongly expressed in the tentacle buds, siphonoglyph and aboral epidermis. (E-E’) *cv2* is strongly and (F-F’) *admp* is weakly expressed in a cross-like pattern between the tentacles and then in eight epidermal stripes – a pattern identical to the epidermal pSMAD1/5 activity at this stage (see A-A’ and (26)). (G-H’) pSMAD1/5 activity in juvenile polyps (~ 6 weeks old), (G-G’) lateral view of the head region, intertentacular and epidermal pSMAD1/5 activity indicated by white arrowheads, (H–H’) oral view of the head region, intertentacular pSMAD1/5 activity is visible between individual tentacles (white arrowheads), unspecific, non-nuclear signal is visible in the cavity of individual tentacles (hollow white arrowheads); tb – tentacle bud, si – siphonoglyph, te – tentacle, ph – pharynx. All stainings were performed at least two times independently with 3 or more animals imaged. Scale bars 50 µm (white) and 100 µm (black). Additional File 1: Fig. S4 – Unspecific staining of spirocytes and putative muco-glandular cells by pSMAD1/5 antibody. (A-A’) cross-section of a tentacle (te) shows unspecific staining of the coiled thread and capsule wall in the spirocytes (B-B’) close-up of the cnidoglandular tract (cgt) shows unspecific, cytoplasmic signal in putative muco-glandular cell types. All stainings were performed at least two times independently with 3 or more animals imaged. Scale bar 25 µm. Additional File 1: Fig. S5 – Dynamics of mCherry expression in the *bmp2/4::mCh *transgenic reporter line. *bmp2/4::mCherry* expression (yellow) is bilaterally symmetric in the 2d planula larva (A). At primary polyp stage, it is confined to the mesentery gastrodermis (B). Additional aboral expression domain forms in late planula (C, also detectable on B; See also Figure S3 for in situ hybridization). In the adult, expression is observed in the mesentery gastrodermis. epi – epidermis, mes – mesentery, sf—septal filament. All stainings were performed at least two times independently with 3 or more animals imaged. Scale bars 50 µm. Additional File 1: Fig. S6 – Overview of BMP signaling domains in the mesentery across optical sections. (A-C’’) show different optical sections of the same mesentery region in a longitudinal side view. A’’-C’’ shows the same images as A’-C’ but using a color gradient LUT making the differences in the pSMAD1/5 staining intensity more obvious. All stainings were performed at least two times independently with 3 or more animals imaged. Scale bars 50 µm. rmr—retractor muscle region, mm—medial mesentery, cgt—cnidoglandular tract, ct – ciliated tract. Additional File 1: Fig. S7 – Expression of BMP ligands and receptors in the mesentery. (A) Schematic of a longitudinal section of an adult *Nematostella* polyp and (A’) female and (A’’) male mesentery whole-mounts after dissection, dashed line (purple) indicates the dissection site located between the mesentery stalk (not visible) and the retractor muscle region (rmr). (B-G’) Whole mount tissue pieces of individual female and male mesenteries stained by in situ hybridization for the expression of (B-B’) *bmp2/4*, (C–C’) *bmp5-8*, (D-D’) *alk2*, (E-E’) *alk6*, (F-F’) *acrRII* and (G-G’) *bmpRII*. All stainings were performed at least two times independently with 3 or more animals imaged. Scale bar 100 µm. phr – pharynx region, rmr – retractor muscle region, cgt – cnidoglandular tract, ct—ciliated tract, oo—oocytes, sp – spermaries. Additional File 1: Fig. S8 – Expression dynamics of BMP pathway genes during embryonic development of *Nematostella*. Expression of (A) *bmp2/4*, *bmp5-8*, *gdf5-l*, *admp*, (B) *alk2*, *alk6*, *actRII* and *bmpRII*, (C) *smad1/5* and *smad4* in the egg and during early stages of *Nematostella* development according to the NvERTx database [43–45]. Additional File 1: Fig. S9 – Differential cadherin localization in the mesentery. (A-A’) Broad localization of cdh3 throughout the gastrodermis and distinct localization of cdh1 localization in the septal filament (ingrowth of the pharyngeal tissue [89]; and (B) in the accessory cells. All stainings were performed at least two times independently with 3 or more animals imaged. Scale bars 50 µm. Additional File 1: Fig. S10 – No detection of pSMAD1/5 in several medusozoan cnidarian species. (A’-F’) show no or non-nuclear signal. All stainings were performed two times independently with 5 or more animals imaged. Scale bar 50 µm. Additional File 1: Fig. S11 – *bmp2/4* knockout animals sporadically develop tentacle-like bubbles. (A) Results of the genotyping of a mosaic F0 mutant and of a F1 heterozygous mutant animal with a single G deletion generating a frame shift. (B-C) Heterozygous *bmp2/4* mutants with a normal morphology (B) and with multiple bubbles (C). (D-E’) Morphological features of the bubble. (D) Phalloidin staining of the bubble tissue shows epidermal muscle fibers exclusive for the normal tentacle tissue. (E-E’) Differential interference contrast image of the same area as on (D) shows multiple spirocytes (arowheads) – a cnidocyte type unique for the tentacles. (F) qPCR comparison of the bodywall marker *cdh1* and tentacle markers *cdh3* and *nem64* in wild-type bodywall (wt_bw) tissue and bubble tissue of the F0 mosaic *bmp2/4* mutants. (G) Overview of morphological and molecular differences between the adult wt polyp and the F0 mosaic *bmp2/4* mutant. Scale bars 5 mm (B-C) and 50 µm (E). epi – epidermis, gastro – gastrodermis, mg – mesoglea (yellow shading), em – epidermal muscle (blue shading).

## Data Availability

All data generated or analysed during this study are included in this published article, its supplementary information files and publicly available repositories. R scripts used for the analysis are available on GitHub (https://github.com/technau/Nv2_Atlas, https://github.com/technau/BMPatlas/releases/tag/Vs.BioRxiv) and Zenodo (10.5281/zenodo.14765821).

## Abbreviations

ac: Accessory cells
bwe: Body wall epidermis
bwg: Body wall gastrodermis
cd: Central disc
ct: Ciliated tract
cgt: Cnidoglandular tract
epi: Epidermis
em: Epidermal muscle
gd: Gastrodermis
gf: Gastric filament
gfb: Gastric filament base
gon: Gonad
gv: Germinal vesicle
itr: Inter-tentacular region
mla: Marginal lappet
ma: Manubrium
mes: Mesentery
mg: Mesoglea
mo: Mouth
mm: Medial mesentery
ms: Mesentery stalk
nc: Nematocyst wart
oo: Oocyte
obw: Outer body wall
pe: Pedalium
pha: Pharynx
pm: Parietal muscle
pmr: Parietal muscle region
rm: Retractor muscle
rmr: Retractor muscle region
rt: Reticular tract
sgg: Somatic gonad gastrodermis
si: Siphonoglyph
sf: Septal filament
sg: Somatic gonad
si: Siphonoglyph
sp: Spermary
spc: Spermatocytes
spg: Spermatogonia
spt: Spermatids and sperm
tb: Tentacle base
tb-ep: Tentacle base epidermis
tb-gd: Tentacle base gastrodermis
te: Tentacle
trg: Trophic gastrodermis
um: Umbrella
vel: Velarium
vla: Velar lappet

## Footnotes

1 In cnidarian developmental biology literature, the terms „ectoderm “, „(mes)endoderm “, and “pharyngeal ectoderm” are used to describe embryonic tissues. Since this paper primarily deals with adult tissues, we will be using the morphological terms „epidermis “ for the outer cell layer and „gastrodermis “ for the lining of the gut. We will also extrapolate the use of these terms to embryonic and larval tissues.

2 Scyphozoa, Cubozoa and Staurozoa all have radially symmetric body plans, however, in Hydrozoa, there are known exceptions. Few hydrozoan species display a clear bilaterality, with an additional plane of symmetry created by the tentacle arrangement in the polyp or the medusa [90], and in the case of the colonial siphonophores, by the distinct functional specialization of the units of the colony (zooids), but also in the embryo [91]. To this point, it is not clear if the secondary body axes of Hydrozoa and Anthozoa are homologous or evolved independently.
