# Supplementary material for: A whole-body atlas of BMP signaling activity in an adult sea anemone
Source: BMC Biol. 2025 Feb 21;23:49. doi: 10.1186/s12915-025-02150-w (PMC11846459; doi:10.1186/s12915-025-02150-w)
Supplement: Supplementary file 1 — Additional file 1. Fig. S1 – Phylogenetic analyses of Nematostella TGFβ receptors and CV2. Maximum likelihood phylogenies were constructed using IQ-tree [88], the bootstrap values are indicated at the nodes. Protein IDs used in the tree are listed in the Methods. Additional File 1: Fig. S2 – Dot plot showing the expression of BMP pathway genes in single-cell transcriptomic data of adult Nematostella polyp tissues with fine clustering presenting 91 transcriptomic cell states (36). Percent Expressed – dot size indicates the percentage of cells in a cluster expressing the gene of interest, Average Expression – color indicates averaged expression value. Additional File 1: Fig. S3 – BMP signaling activity and expression of BMP signaling components in the head region. (A-A’) pSMAD1/5 activity between the forming tentacles and around the mouth of the 4d planula. (B) bmp2/4 expression in the oral gastrodermis and in the aboral epidermis of the planula; faint expression is discernable in the tentacle buds, (C) bmp5-8 is also expressed in the gastrodermis, tentacle buds and aboral epidermis, but appears stronger than bmp2/4. (D-D’) rgm is strongly expressed in the tentacle buds, siphonoglyph and aboral epidermis. (E-E’) cv2 is strongly and (F-F’) admp is weakly expressed in a cross-like pattern between the tentacles and then in eight epidermal stripes – a pattern identical to the epidermal pSMAD1/5 activity at this stage (see A-A’ and (26)). (G-H’) pSMAD1/5 activity in juvenile polyps (~ 6 weeks old), (G-G’) lateral view of the head region, intertentacular and epidermal pSMAD1/5 activity indicated by white arrowheads, (H–H’) oral view of the head region, intertentacular pSMAD1/5 activity is visible between individual tentacles (white arrowheads), unspecific, non-nuclear signal is visible in the cavity of individual tentacles (hollow white arrowheads); tb – tentacle bud, si – siphonoglyph, te – tentacle, ph – pharynx. All stainings were performed at least two times independent [file 12915_2025_2150_MOESM1_ESM.pdf]

Supplementary Figures

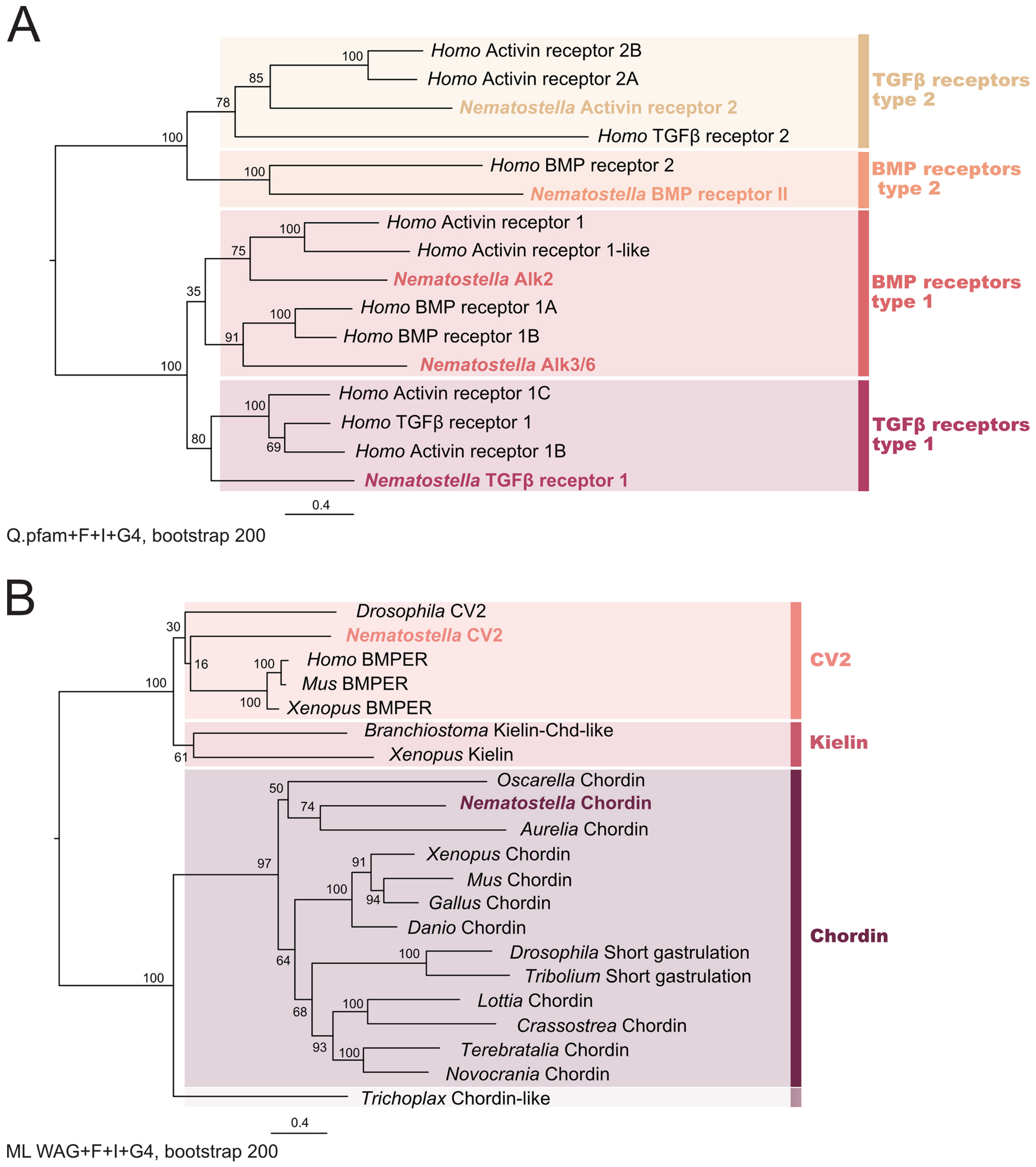

Figure S1. Phylogenetic analyses of *Nematostella* TGFβ receptors and CV2. Maximum likelihood phylogenies were constructed using IQ-tree (90), the bootstrap values are indicated at the nodes. Protein IDs used in the tree are listed in the Methods.

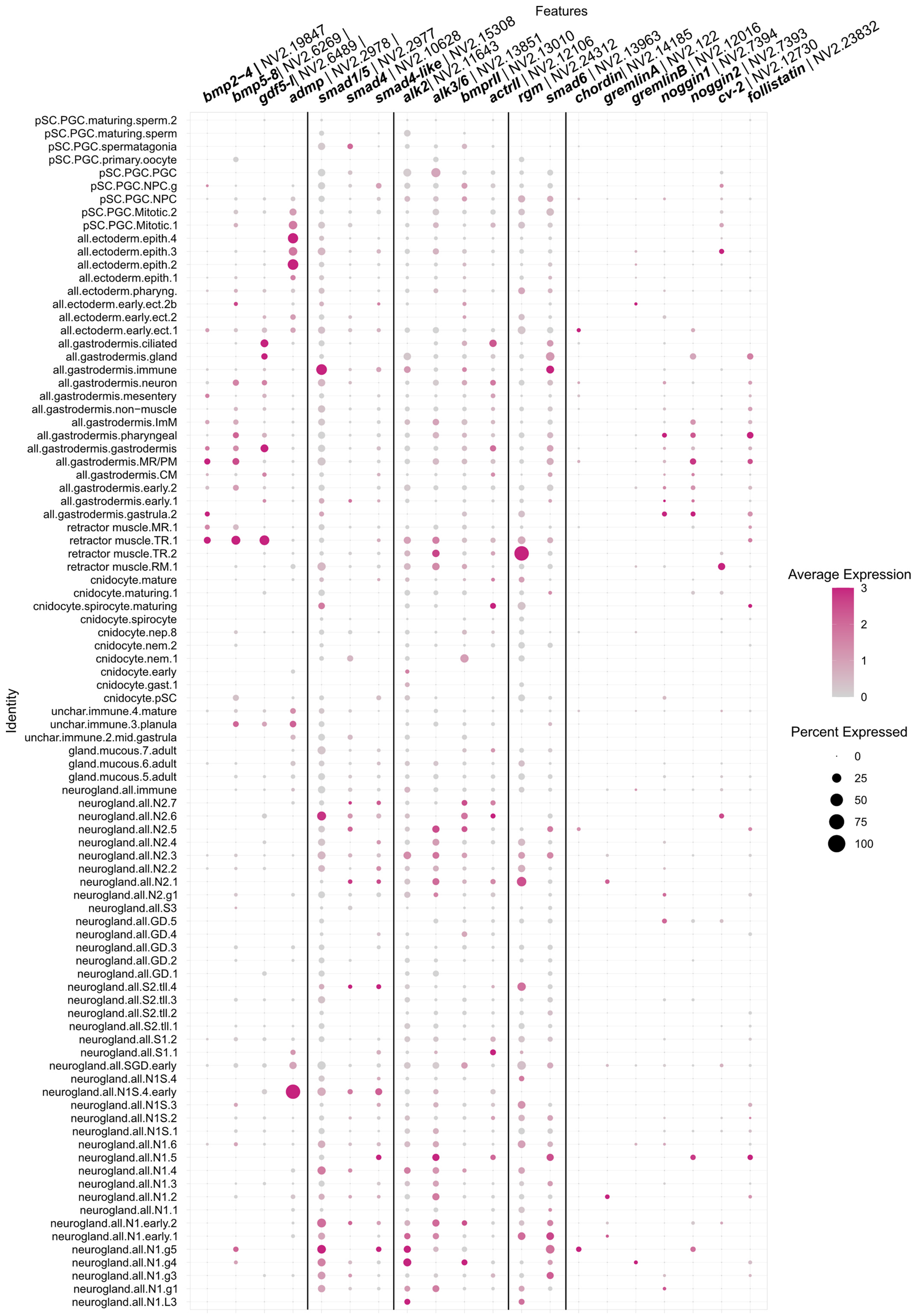

Figure S2. Expression of BMP pathway genes in single-cell transcriptomic data of adult *Nematostella* polyp tissues with fine clustering presenting 91 transcriptomic cell states (36). Percent Expressed – dot size indicates the percentage of cells in a cluster expressing the gene of interest, Average Expression – color indicates averaged expression value.

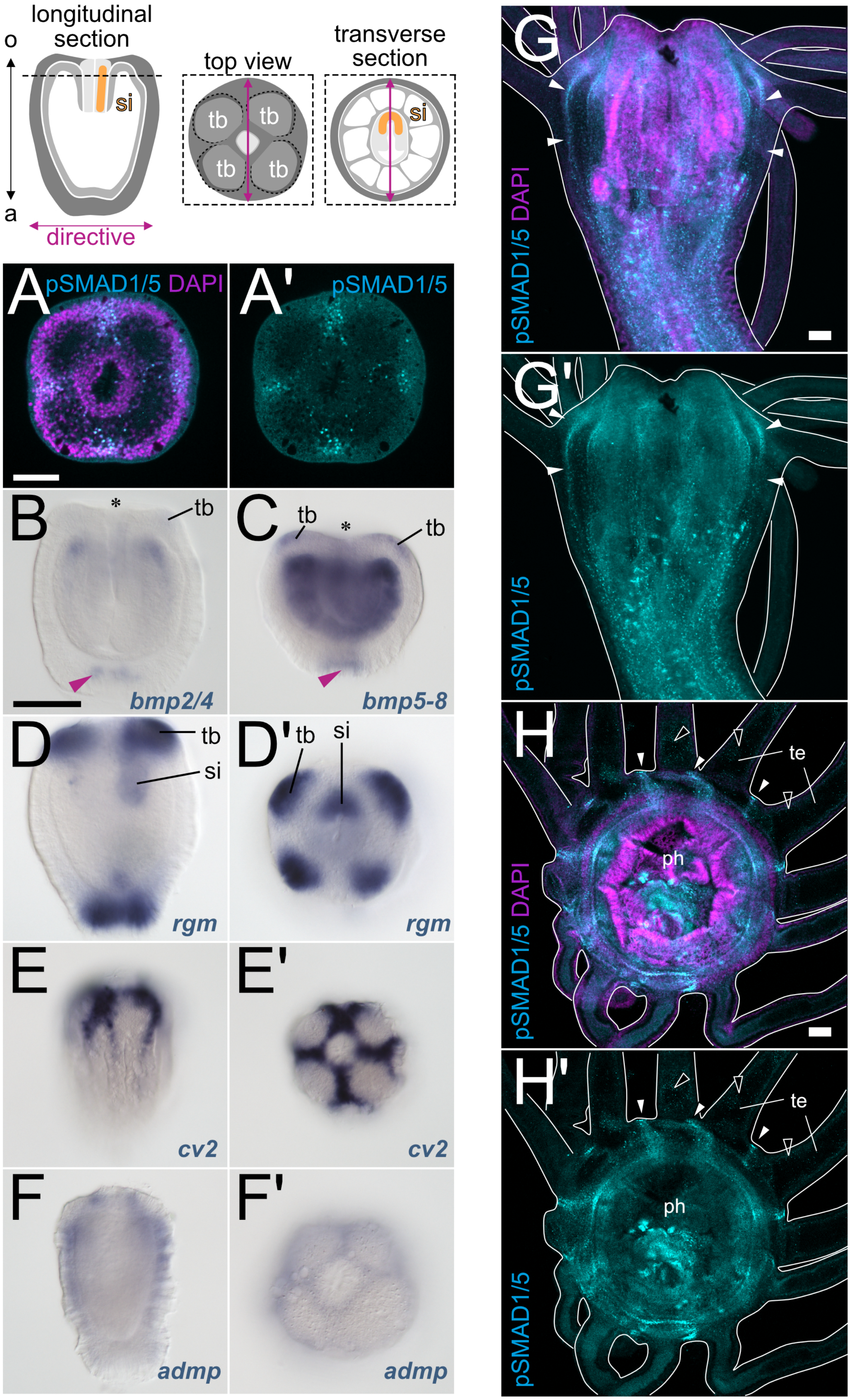

Figure S3. BMP signaling activity and expression of BMP signaling components in the head region. (A-A') pSMAD1/5 activity between the forming tentacles and around the mouth of the 4d planula. (B) *bmp2/4* expression in the oral gastrodermis and in the aboral epidermis of the planula; faint expression is discernable in the tentacle buds, (C) *bmp5-8* is also expressed

Figure S3 (continued).
in the gastrodermis, tentacle buds and aboral epidermis, but appears stronger than *bmp2/4*. (D-D’) *rgm* is strongly expressed in the tentacle buds, siphonoglyph and aboral epidermis. (E-E’) *cv2* is strongly and (F-F’) *admp* is weakly expressed in a cross-like pattern between the tentacles and then in eight epidermal stripes – a pattern identical to the epidermal pSMAD1/5 activity at this stage (see A-A’ and (26)). (G-H’) pSMAD1/5 activity in juvenile polyps (~6 weeks old), (G-G’) lateral view of the head region, intertentacular and epidermal pSMAD1/5 activity indicated by white arrowheads, (H-H’) oral view of the head region, intertentacular pSMAD1/5 activity is visible between individual tentacles (white arrowheads), unspecific, non-nuclear signal is visible in the cavity of individual tentacles (hollow white arrowheads); tb – tentacle bud, si – siphonoglyph, te – tentacle, ph – pharynx. All stainings were performed at least two times independently with 3 or more animals imaged. Scale bars 50 µm (white) and 100 µm (black).

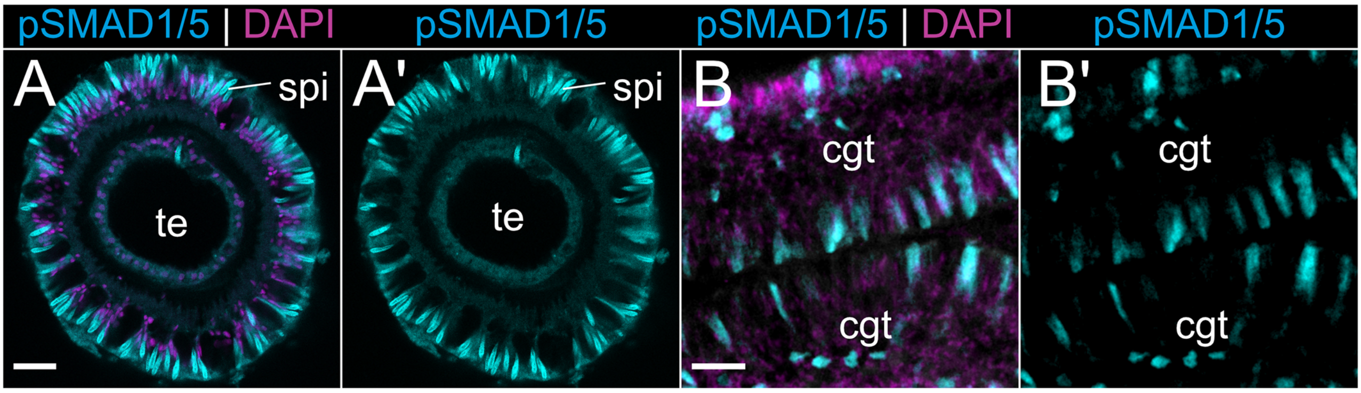

Figure S4. Unspecific staining of spirocytes and mucoglandular cells by pSMAD1/5 antibody. (A-A’) cross-section of a tentacle (te) shows unspecific staining of the coiled thread and capsule wall in the spirocytes (B-B’) close-up of the cnidoglandular tract (cgt) shows unspecific, cytoplasmic signal in muco-glandular cell types. All stainings were performed at least two times independently with 3 or more animals imaged. Scale bar 25 µm.

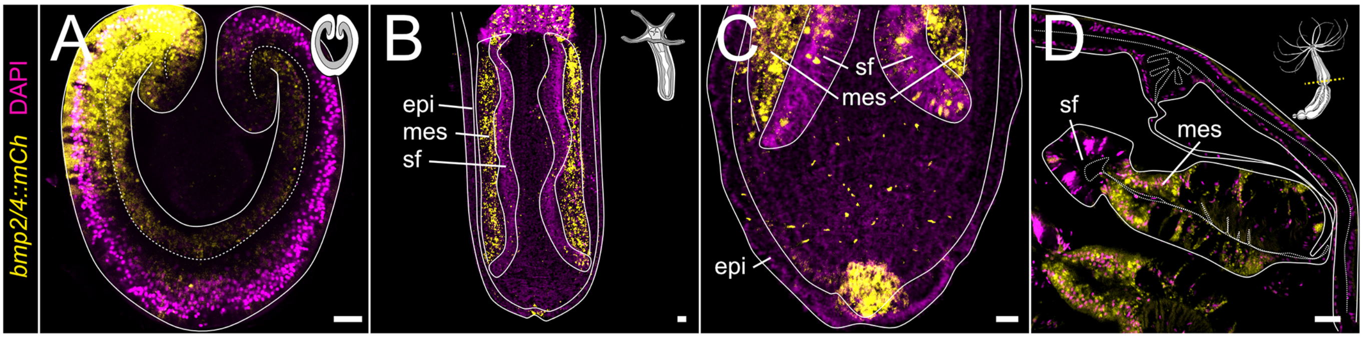

Figure S5. Dynamics of mCherry expression in the *bmp2/4::mCh* transgenic reporter line. *bmp2/4::mCherry* expression (yellow) is bilaterally symmetric in the 2d planula larva (A). At primary polyp stage, it is confined to the mesentery gastrodermis (B). Additional aboral expression domain forms in late planula (C, also detectable on B; See also Figure S3 for in situ hybrdization). In adult, expression is observed in the mesentery gastrodermis. epi – epidermis, mes – mesentery, sf - septal filament. All stainings were performed at least two times independently with 3 or more animals imaged. Scale bars 50 µm.

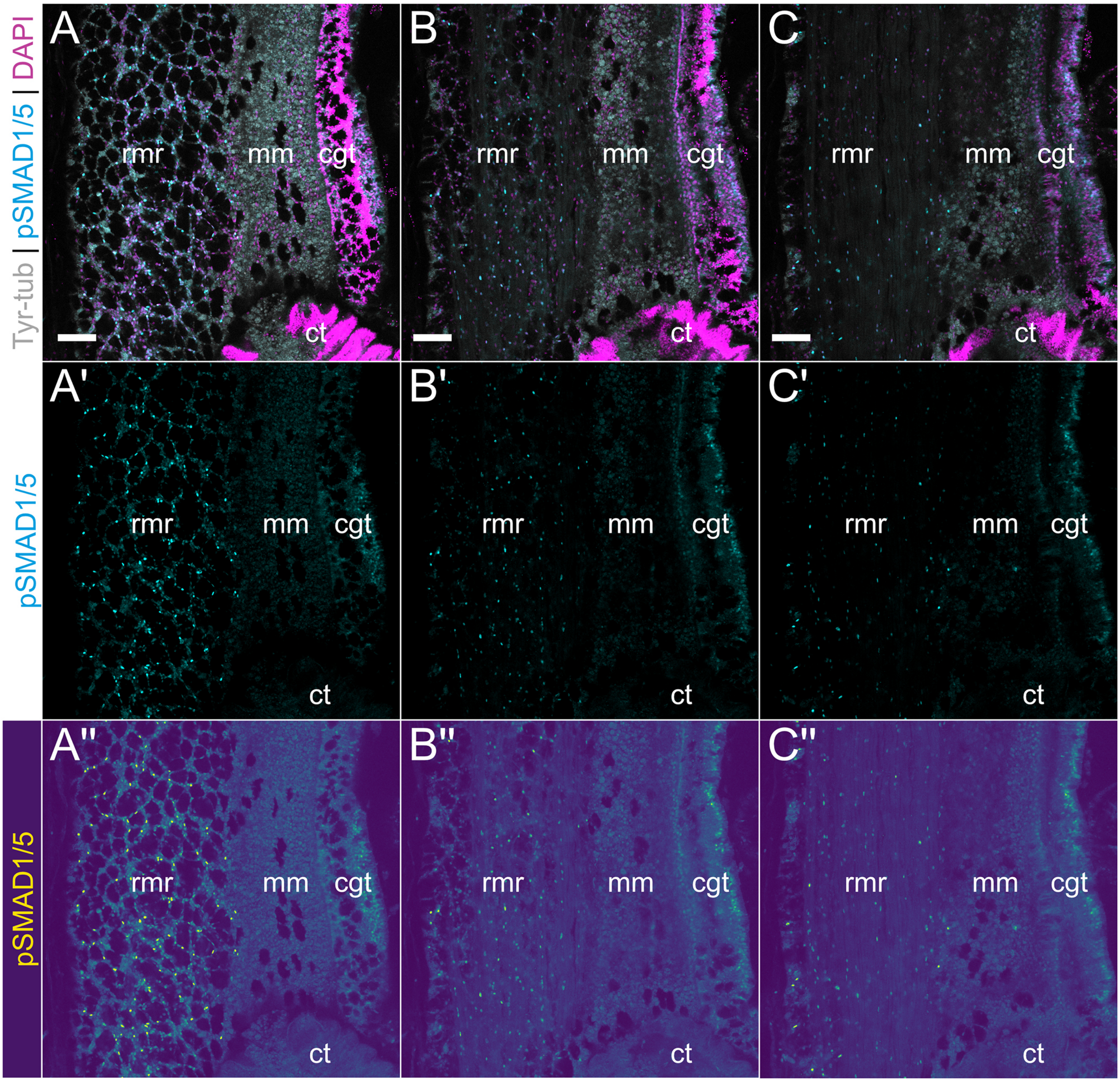

Figure S6. Overview of BMP signaling domains in the mesentery across optical sections. (A-C'') show different optical sections of the same mesentery region in a longitudinal side view. A''-C'' shows the same images as A'-C' but using a color gradient LUT making the differences in the pSMAD1/5 staining intensity more obvious. All stainings were performed at least two times independently with 3 or more animals imaged. Scale bars 50 µm. rmr - retractor muscle region, mm - medial mesentery, cgt - cnidoglandular tract, ct – ciliated tract.

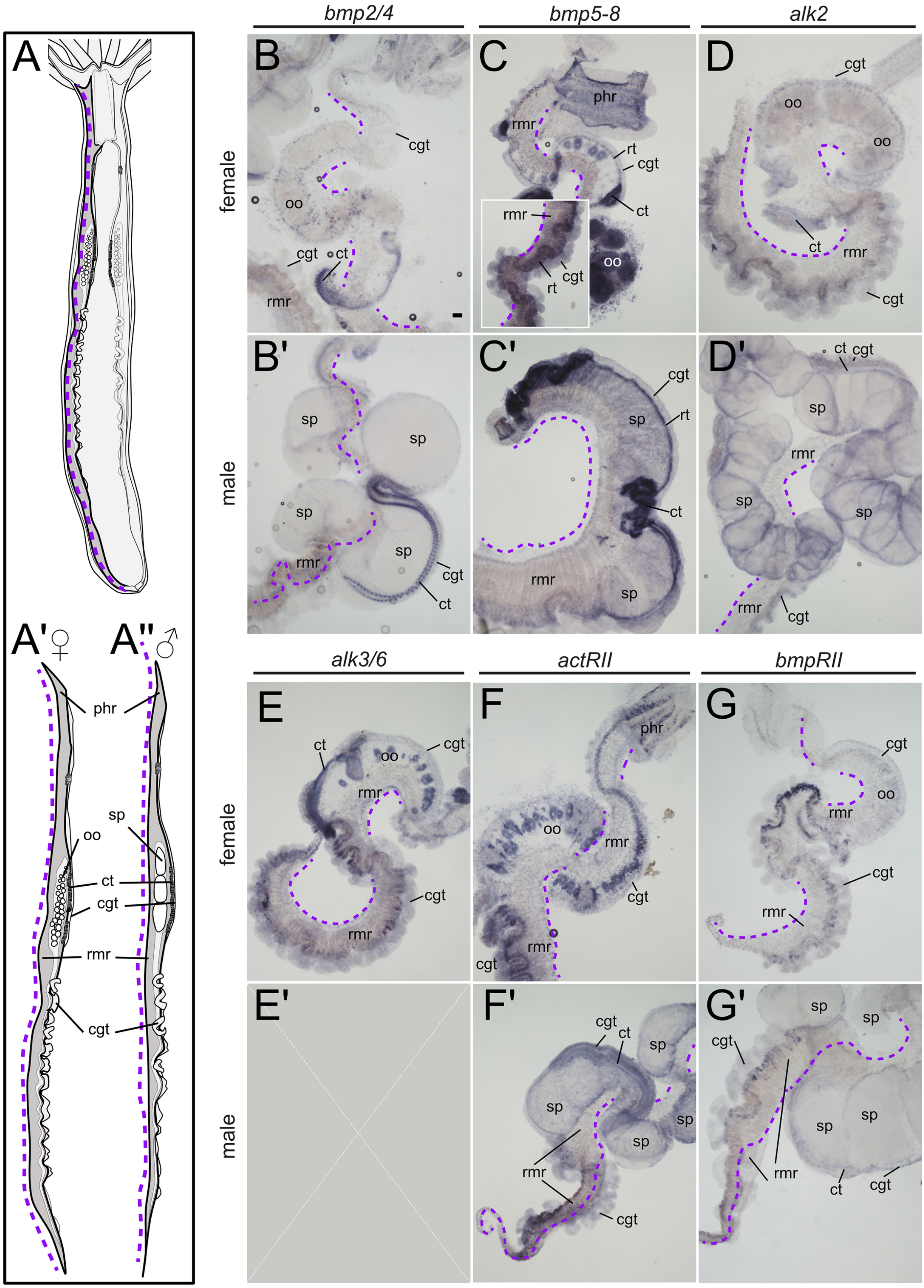

Figure S7. Expression of BMP ligands and receptors in the mesentery. (A) Schematic of a longitudinal section of an adult *Nematostella* polyp and (A') female and (A'') male mesentery whole-mounts after dissection, dashed line (purple) indicates the dissection site located between the mesentery stalk (not visible) and the retractor muscle region (rmr). (B-G') Whole mount tissue

Figure S7 (continued).
pieces of individual female and male mesenteries stained by ISH for the expression of (B-B’) bmp2/4, (C-C’) bmp5-8, (D-D’) alk2, (E-E’) alk6, (F-F’) acrRII and (G-G’) bmpRII. All stainings were performed at least two times independently with 3 or more animals imaged. Scale bar 100 µm. phr – pharynx region, rmr – retractor muscle region, cgt – cnidoglandular tract, ct - ciliated tract, oo - oocytes, sp – spermaries.

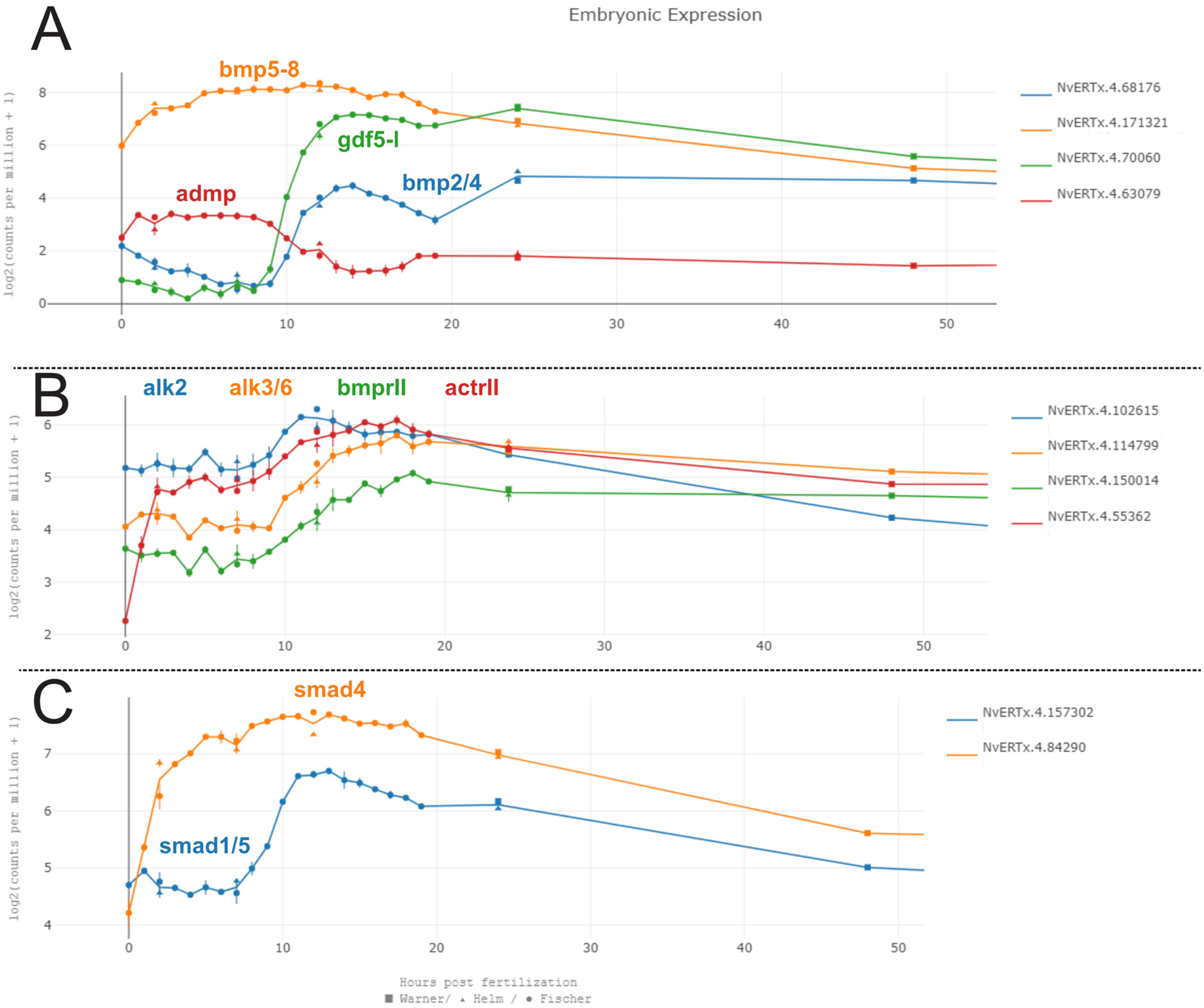

Figure S8. Expression dynamics of BMP pathway genes during embryonic development of *Nematostella*. Expression of (A) *bmp2/4*, *bmp5-8*, *gdf5-l*, *admp*, (B) *alk2*, *alk3/6*, *actRII* and *bmpRII*, (C) *smad1/5* and *smad4* in the egg and during early stages of *Nematostella* development according to the NvERTx database (45-47).

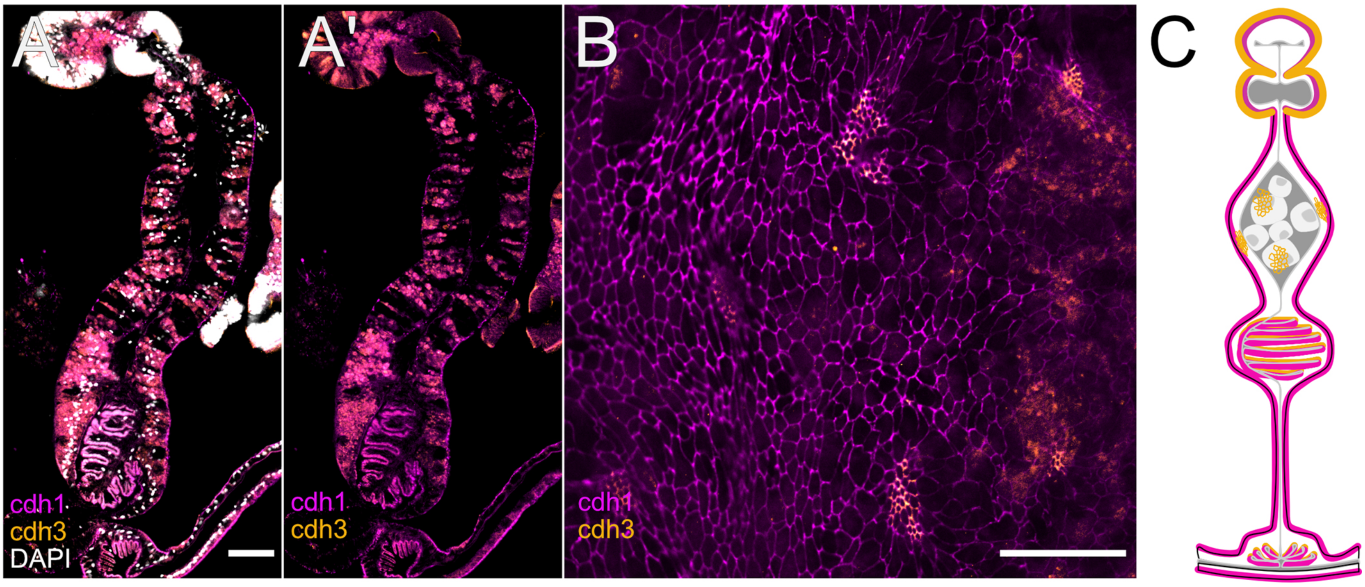

Figure S9. Differential cadherin localization in the mesentery. (A-A') Broad localization of cdh3 throughout the gastrodermis and distinct localization of cdh1 localization in the septal filament (ingrowth of the pharyngeal tissue (91)) and (B) in the accessory cells. All stainings were performed at least two times independently with 3 or more animals imaged. Scale bars 50 μm.

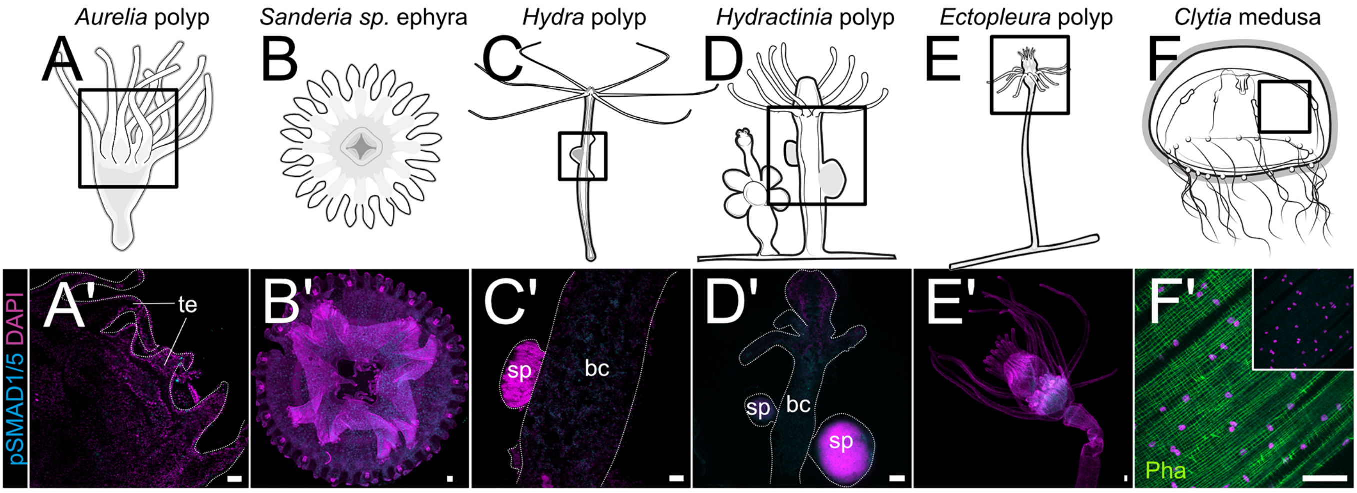

Figure S10. No detection of pSMAD1/5 in several medusozoan cnidarian species. (A'-F') show no or non-nuclear signal. All stainings were performed at least two times independently with 3 or more animals imaged. Scale bar 50 µm.

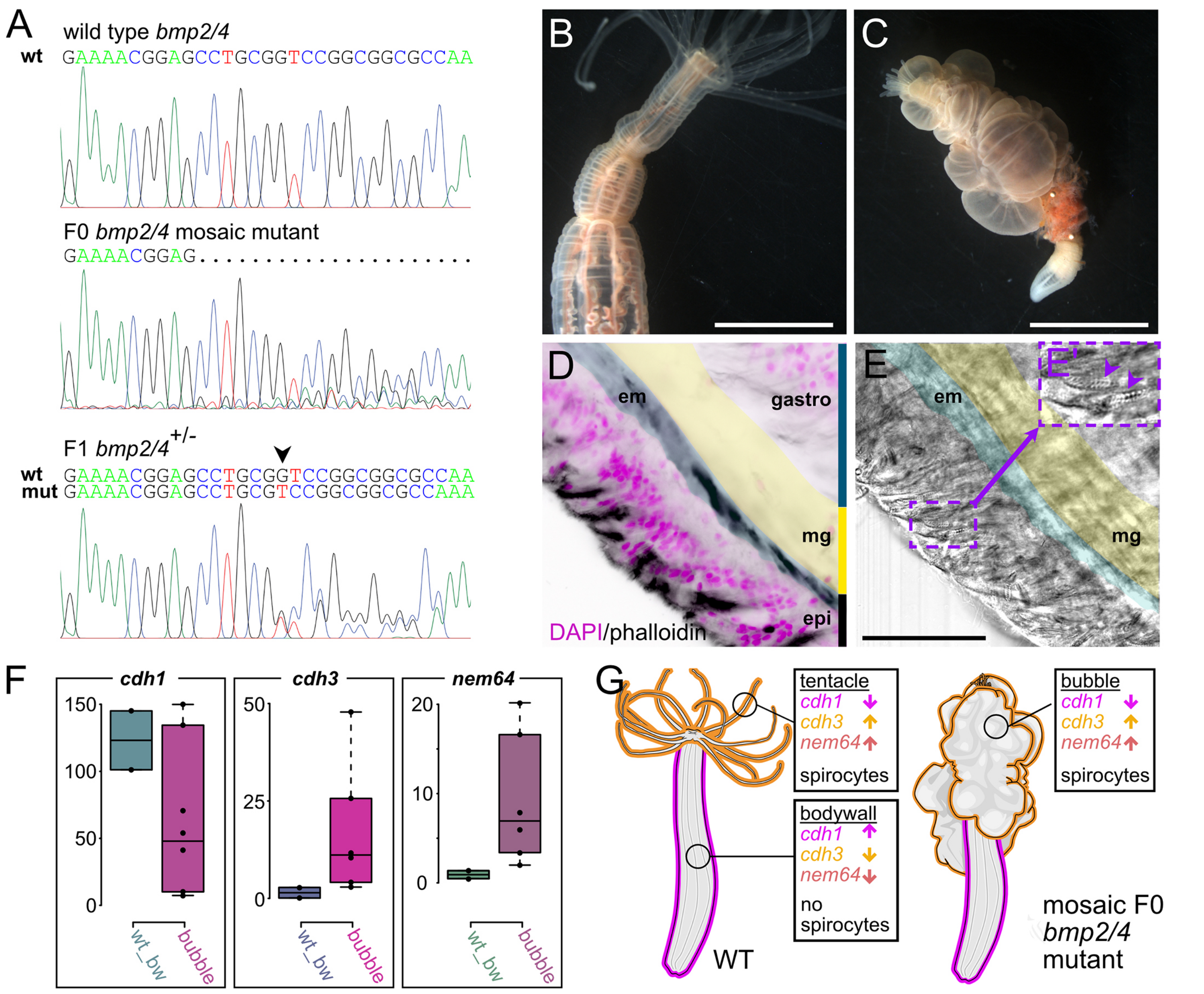

Figure S11. ***bmp2/4*** **knockout animals sporadically develop tentacle-like bubbles.**
(A) Results of the genotyping of a mosaic F0 mutant and of a F1 heterozygous mutant animal with a single G deletion generating a frame shift. (B-C) Heterozygous *bmp2/4* mutants with a normal morphology (B) and with multiple bubbles (C). (D-E') Morphological features of the bubble. (D) Phalloidin staining of the bubble tissue shows epidermal muscle fibers exclusive for the normal tentacle tissue. (E-E') Differential interference contrast image of the same area as on (D) shows multiple spirocysts (arowheads) – a cnidocyte type unique for the tentacles. Scale bars 5 mm (B-C) and 50 µm (E). epi – epidermis, gastro – gastrodermis, mg – mesoglea (yellow shading), em – epidermal muscle (blue shading). (F) qPCR comparison of the bodywall marker *cdh1* and tentacle markers *cdh3* and *nem64* in wild-type bodywall (wt_bw) tissue and bubble tissue of the F0 mosaic *bmp2/4* mutants. (G) Overview of morphological and molecular differences between the adult wt polyp and the F0 mosaic *bmp2/4* mutant. Scale bars 5 mm (B-C) and 50 µm (E). epi – epidermis, gastro – gastrodermis, mg – mesoglea (yellow shading), em – epidermal muscle (blue shading).
